# A comprehensive benchmark of graph-based genetic variant genotyping algorithms on plant genomes for creating an accurate ensemble pipeline

**DOI:** 10.1186/s13059-024-03239-1

**Published:** 2024-04-08

**Authors:** Ze-Zhen Du, Jia-Bao He, Wen-Biao Jiao

**Affiliations:** 1 National Key Laboratory for Germplasm Innovation & Utilization of Horticultural Crops, Huazhong Agricultural University, Wuhan, China; 2 Hubei Hongshan Laboratory, Wuhan, China

**Keywords:** Genome graph, Plant genomes, Genotyping, Structural variation, Benchmarking

## Abstract

**Background:**

Although sequencing technologies have boosted the measurement of the genomic diversity of plant crops, it remains challenging to accurately genotype millions of genetic variants, especially structural variations, with only short reads. In recent years, many graph-based variation genotyping methods have been developed to address this issue and tested for human genomes. However, their performance in plant genomes remains largely elusive. Furthermore, pipelines integrating the advantages of current genotyping methods might be required, considering the different complexity of plant genomes.

**Results:**

Here we comprehensively evaluate eight such genotypers in different scenarios in terms of variant type and size, sequencing parameters, genomic context, and complexity, as well as graph size, using both simulated and real data sets from representative plant genomes. Our evaluation reveals that there are still great challenges to applying existing methods to plants, such as excessive repeats and variants or high resource consumption. Therefore, we propose a pipeline called Ensemble Variant Genotyper (EVG) that can achieve better genotyping performance in almost all experimental scenarios and comparably higher genotyping recall and precision even using 5× reads. Furthermore, we demonstrate that EVG is more robust with an increasing number of graphed genomes, especially for insertions and deletions.

**Conclusions:**

Our study will provide new insights into the development and application of graph-based genotyping algorithms. We conclude that EVG provides an accurate, unbiased, and cost-effective way for genotyping both small and large variations and will be potentially used in population-scale genotyping for large, repetitive, and heterozygous plant genomes.

**Supplementary Information:**

The online version contains supplementary material available at 10.1186/s13059-024-03239-1.

## Background

Genetic variants are typically divided into single nucleotide polymorphism (SNP), insertion or deletion (indels, 1–49 bp), and structural variation (SV, ≥ 50 bp, including insertion, deletion, inversion, duplication, translocation, and complex rearrangements) based on their size and type [1, 2]. With the advances of high-throughput sequencing technologies, studies such as the 1000 Genomes Project and the Rice 3K Genomes Project have released large amounts of genetic variations, which contribute to the studies of pan-genomes, genome-wide associations, population genetics, and domestication [3–6]. One of the essential requirements for these studies is the rapid and correct genotyping (determination of genotypes) of millions of genetic variations for hundreds or thousands of individuals [3–6]. Conventional genotyping strategies usually rely on short-read mapping against a linear reference genome [2, 7–9]. However, these methods often introduce alignment errors due to reference bias, leading to erroneous genotypes for some variants, particularly those from regions highly divergent from the reference [10, 11]. In particular, genotyping SVs remains extremely challenging in population-scale studies where many individual genomes are sequenced solely using short-read sequencing technologies [3, 5, 11].

Recent advancements in pangenome graph-based genotyping algorithms are expected to mitigate reference bias and enhance the accuracy of genotyping across all types of genetic variations [12–15]. In such a graph, nodes typically represent the sequences, and edges indicate the connections between the sequences. Variations manifest as “bubbles”, and a path through the graph can be transformed into a haplotype sequence that represents a combination of different sequence variations [15]. By incorporating the reference genome as well as non-reference alleles into sequence or variation graphs, these algorithms can precisely genotype variations for individual genomes based on short-read data. They use either read (e.g., vg and GraphTyper2) or k-mer (e.g., BayesTyper and PanGenie) alignments against the graphs to achieve high accuracy [16–19]. However, the complex pangenome graph also expands the search space for read mapping. For instance, the original vg algorithm (vg map) maps short reads to arbitrary variation graphs using generalized compressed suffix arrays to remove reference bias and improve alignment accuracy. Nevertheless, it is at least an order of magnitude slower than linear genome mappers, making it challenging to apply to large genomes or complex graphs [16]. A more recent version, vg giraffe, based on the seed-and-extend algorithm, can accelerate mapping [20]. Unlike vg map and vg giraffe, which map reads to the whole-genome graph, only aligning reads to variant breakpoints (such as GraphTyper2) or comparing read k-mer coverages at k-mer represented variants (such as BayesTyper and PanGenie) can also reduce runtime [17–19]. Besides, the mapping accuracy of reads may decrease as the number of nodes (variants) in the graph increases [21].

Remarkably, most of these algorithms were initially developed and tested for human genomes [16–18, 20, 22–24]. Although some graph-based genotypers such as vg have been applied to variation genotyping for crop genomes like rice [25], soybean [26], tomato [27], etc., the detailed performance of these tools remains elusive for plants as the complexity of plant genomes varies greatly in terms of genome size, repeat content, heterozygosity, and polyploidy. For example, repeat-enriched SVs can introduce inaccurate coverages of k-mers or reads at variant sites, thereby affecting the performance of graph-based genotyping methods that rely on such coverages for genotyping [18, 19, 23].

To address these issues, we first investigate the impact of read length and depth, number of variants, repeat density, heterozygous rate, etc. on existing graph-based genotypers in plant genomes [16–20, 24, 28]. Our findings suggest that there are still some challenges in applying existing methods to plants, such as worse performance with excessive repeats and variants or high resource consumption. To overcome these challenges, we present an Ensemble Variant Graph-based tool, EVG, which can accurately genotype SNPs, indels, and SVs using short reads. Compared to other graph-based genotypers, EVG achieves higher genotyping accuracy and recall with only 5× sequencing data. Furthermore, the genotyping of EVG remains robust even as the number of nodes in the pangenome graph increases.

## Results

### Graph-based variant genotyper selection

To our knowledge, there are currently twelve graph-based genotyping tools available (Additional file 1: Table S1). For this study, we selected eight open-source graph-based genotyping tools that broadly fall into two categories: read alignment based (including vg map [16], vg giraffe [20], Paragraph [24], GraphTyper2 [17] and Gramtools [29]) and k-mers alignment based (including BayesTyper [18] and PanGenie [19]) (Additional file 1: Table S1). We also conducted experiments to assess the performance of GraphAligner [28], a graph-based aligner that can utilize graphs constructed by vg to do alignment. Other tools were excluded from this study either because they are currently unsuitable for plant genomes (such as HISAT [30] and Minos [31]) or because they cannot genotype all types of genetic variations (like KAGE [32]). Among them, Minos is designed for bacterial genomes, while HISAT-genotype requires reconstruction of a typing database and complex conversion for plants in the algorithm.

These tools utilize different graph indexing approaches to improve alignment efficiency and/or support multiple graph manipulations (Additional file 1: Table S1). Specifically, vg employs GBWT [33], GCSA2 [34], and Minimizers [35] for graph storage and searching, whereas BayesTyper relies on k-mer-based graph indexing. To avoid potential memory overload, BayesTyper leverages Bloom filters to screen read k-mers, storing only those present in the haplotype [18]. Similarly, PanGenie adopts k-mer-based graph indexing using De Bruijn graphs. Subsequently, the software either aligns reads to nodes or directly counts k-mer coverage at nodes [19]. The genotyping results are then probabilistically scored based on statistical distribution modeling of observed and noise read/k-mer coverages.

### Overall performance on simulated data

To evaluate the performance of the tools, we first constructed a comprehensive simulation panel. Considering that plant genomes vary widely in size and repeats [36, 37], a series of data sets of paired-end shorted reads were simulated for each of five representative plant genomes (*Arabidopsis thaliana*, *Oryza sativa*, *Glycine max*, *Zea mays*, and *Brassica napus*) with different genome sizes (135–2300 Mb) and repeat contents (21.42–88.9%) (Additional file 1: Table S2). To generate simulated short reads from alternative (no-reference) genomes, we introduced different types (SNP, indels (< 50 bp), and SVs (≥ 50 bp)) and numbers of variants into the reference genome of each plant species [5, 26, 38–40] (see Methods for details). We repeat such a simulation of paired-end short reads with varying read lengths (100 bp, 150 bp, 250 bp), insert sizes (300 bp, 400 bp, 500 bp, 600 bp), and sequencing depths (5×, 10×, 20×, 30×, 50×) (Additional file 1: Table S3). Precision and recall were used to assess the genotyping performance of different software, and receiver operating characteristic (ROC) curves were drawn according to the genotype quality (GQ) or read depth (DP).

**Fig. 1:**
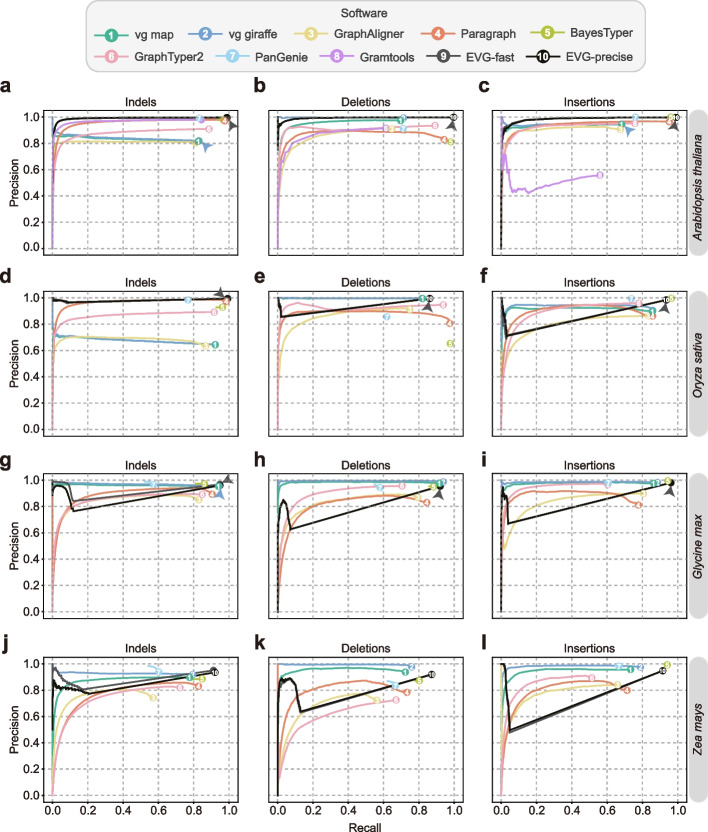
Overall genotyping performance of different graph-based tools based on simulated data. The genome graphs of *Arabidopsis thaliana* (**a**, **b**, **c**), *Oryza sativa* (**d**, **e**, **f**), *Glycine max*, (**g**, **h**, **i**), and *Zea mays* (**j**,** k**, **l**), are constructed based on one reference genome and seven alternative genomes derived by introducing known variants into the reference genome. Paired-end (2 × 150 bp) short reads with 30× depth are simulated for genotyping. For each genotyper, precision is plotted against recall as the genotyping quality threshold varies. Read Depth on variant sites is used as a substitution score when genotype quality is not available. Arrows indicate the circles hidden by other circles in the plot due to identical or nearly identical precision values. Detailed results are also provided in Additional file 2: Table S5

As graph-based genotyping tools can leverage sequence information from multiple genomes, we first determined the overall performance of these tools based on the genome graph from eight *A. thaliana* genomes (one reference genome assembly, and all variants from the other seven genomes) (Fig. 1a–c; Additional file 1: Fig. S1a). For one genome to be genotyped, we simulated 467,512 SNPs, 38,207 indels, 4572 insertions, 4364 deletions, 232 inversions, and 100 duplications (Additional file 1: Table S4). Note that all these variants are incorporated into the genome graph. To genotype this genome using each tool, we simulated 30× paired-end (2 × 150 bp) short reads. For SNP genotyping, all tools demonstrate high precision (> 0.97), while only BayesTyper (0.99), Paragraph (0.98), and GraphTyper2 (0.93) have a recall rate greater than 0.90 (Additional file 1: Fig. S1a). Similarly, nearly all tools show high precision rates (0.80–0.99) and relatively lower recall rates (0.81–0.98) for indels genotyping. However, only BayesTyper (0.98), PanGenie (0.99), Gramtools (0.98), and Paragraph (0.97) maintain precision above 0.95 (Fig. 1a). The performance of genotyping large insertions and deletions (≥ 50 bp) varies greatly among the tools. Despite different recall rates, all tools except Gramtools present genotyping precision above 0.8 (Fig. 1b, c). Overall, Paragraph, GraphTyper2, and BayesTyper outperformed other tools in terms of genotyping performance (Fig. 1b, c).

We also evaluated these tools using the simulated data from rice, soybean, maize, and *Brassica napus* genomes (Additional file 1: Tables S2, S3). Note that Gramtools is excluded from the assessment for other plant genomes due to potential issues related to excessive chromosome length. For the large maize genomes, we conducted our evaluation on chromosome 10 instead of the entire genome to reduce testing time. We observed a similar recall of genotyping all types of variants in rice genomes as in *A. thaliana*, but with slightly decreased precision. (Fig. 1d–f; Additional file 1: Fig. S1c). The genotyping performance in soybean genomes was better than that of maize, but worse than *A. thaliana* and rice genomes (Fig. 1g–i; Additional file 1: Fig. S1e). For genotyping in the maize genome, vg map and vg giraffe are the only tools that maintain high precision (0.93–0.98) and recall (0.72–0.80) in SV genotyping, while BayesTyper and Paragraph present high precision (0.87, 0.83) and recall (0.85, 0.83) for indel genotyping (Fig. 1j–l; Additional file 1: Fig. S1f). When genotyping in the allotetraploid *Brassica napus*, the performance is even worse, especially for SNPs and indels (Additional file 1: Fig. S2). This may be due to the inference of homoeologous alleles between the two subgenomes of *Brassica napus*.

When genotyping complex SVs like inversions and duplications, the performance differences between the software are obvious. Although all software can detect inversion, vg map, vg giraffe, GraphAligner, and Gramtools only worked effectively when the number of genomes was one. On the other hand, Paragraph and BayesTyper demonstrated superior performance with *F*-scores over or around 0.8 when genotyping inversion and duplications in graphs containing multiple *A. thaliana* or rice genomes (Additional file 1: Fig. S3a, b). Apparently, these tools presented lower *F*-scores for genotyping heterozygous SVs, especially the duplications (Additional file 1: Fig. S3).

As a comparison, we also employed three linear reference-based genotypers including GATK [41] and DeepVariant [42] (for both SNPs and indels), and Delly [43] (for SV) to do genotyping using the same simulated short reads dataset. Both GATK and DeepVariant had a *F*-score above 0.9 for SNP genotyping in all plant genomes except the maize, but a relatively lower *F*-score in indel genotyping for the same plant genomes (Additional file 1: Fig. S4a, b). We found that some graph-based tools have higher or very close *F*-scores compared to GATK and DeepVariant, although other tools presented relatively lower *F*-scores. However, the graph-based tools exhibited much better performance compared to the tool Delly, especially for the large insertions (Additional file 1: Fig. S4c, d).

### Performance on plant genomes with different complexity

Moreover, tests conducted across various plant genomes revealed that certain tools, such as GraphAligner and GraphTyper2, exhibited relatively poor performance when dealing with larger genomes. Conversely, BayesTyper were able to retain high precision and recall even when working with more complex genomes (Fig. 1; Additional file 1: Fig. S1; Additional file 2: Table S5).

**Fig. 2:**
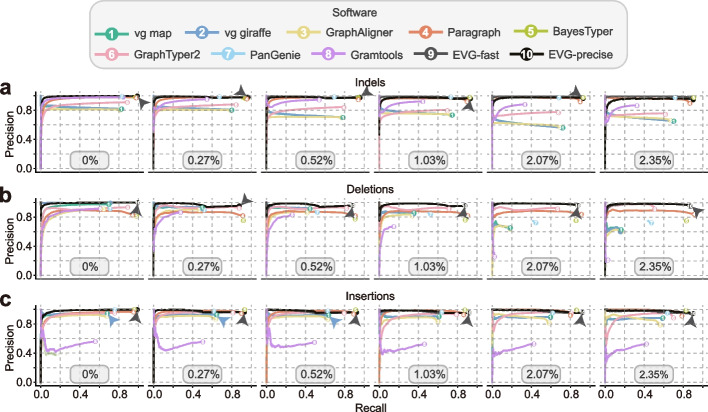
The effect of heterozygous rate on the performance of different graph-based genotyping methods. The six ROC curve plots correspond to the genotyping results for synthetic heterozygous *A. thaliana* genomes with different heterozygous rates (0%, 0.27%, 0.52%, 1.03%, 2.07%, and 2.35%). The genome graph for genotyping is constructed from the *A. thaliana* reference genome and seven alternative genomes. Paired-end (2 × 150 bp) short reads with 30× depth are simulated for genotyping. For each genotyper, precision is plotted against recall as the genotyping quality threshold varies. Read Depth on variant sites is used as a substitution score when genotype quality is not available. Arrows indicate the circles hidden by other circles in the plot due to identical or nearly identical precision values. Detailed results are also provided in Additional file 2: Table S6

As numerous plant genomes are heterozygous, we also simulated heterozygous *A. thaliana* and rice for the same test. Among the tools we evaluated, Paragraph, BayesTyper, and GraphTyper2 were less affected by heterozygosity, whereas other software experienced a decrease in recall for all variants (Additional file 1: Fig. S1, S5). We also explored the influence of heterozygosity on genotyping by testing on synthetic diploid genomes with varying levels of heterozygosity (Fig. 2; Additional file 1: Fig. S6; Additional file 2: Table S6). The results showed that the damage to genotyping was proportional to the level of heterozygosity, especially for deletions and inversions. Paragraph and BayesTyper proved to be the most stable tools, both with high precision (0.75–0.97) and recall (0.79–0.96) for small and large indel genotyping (Fig. 2; Additional file 1: Fig. S6). However, tools such as vg map, vg giraffe, GraphAligner, and Gramtools were relatively more influenced by heterozygosity (Fig. 2; Additional file 1: Fig. S6), especially for genotyping in repetitive regions (Additional file 1: Fig. S7b).

### Influence of sequencing parameters

Next, we conducted a comparison of each genotyper’s performance across datasets of paired-end reads with a range of read lengths (100 bp, 150 bp, 250 bp), fragment size (300 bp, 400 bp, 500 bp, 600 bp), and sequencing depths (5×, 10×, 20×, 30×, 50×). When the read length was shorter, such as 100 bp, only Paragraph showed a similarly high *F*-score for both small and large variants compared to testing with longer reads (Additional file 1: Fig. S8). Other tools, such as vg map and PanGenie, also had close *F*-scores with shorter (100 bp) or longer reads (150 bp, 250 bp) for variants except inversions. Additionally, a marginal effect could be observed when increasing the read length from 150 to 250 bp (Additional file 1: Fig. S8). Apparently, various types of variants had similar requirements for sequencing length (Additional file 1: Fig. S8). The performance on rice and heterozygous *Arabidopsis* genomes followed the same trend as *A. thaliana*, suggesting that genome size and complexity may not affect the read length requirement by software (Additional file 1: Fig. S9, S10). In addition to using short reads, we also tested GraphAligner to map third-generation data against the genome graph, using long reads of 20 kb and 75 kb. The genotyping accuracy of 20 kb reads was superior to that of 75 kb reads, likely due to the former’s higher accuracy (0.96 vs. 0.85) (Additional file 1: Fig. S8, S10). Furthermore, when the read length was 150 bp, fragment sizes (300–600 bp) had no obvious effect on the genotyping accuracy of these genotypers (Additional file 1: Fig. S11).

Overall, when the sequencing depth was around 5–10×, Paragraph was able to achieve relatively high performance (precision > 0.81, recall > 0.91), whereas other software required more than 10× reads (Additional file 1: Fig. S12). Besides, increasing the sequencing depth beyond 20×, only brought marginal improvements in genotyping performance across all variant types. With 30× data, all tested software almost reached the upper limit of genotyping precision and recall (Additional file 1: Fig. S12). These findings were consistent across different genomes, including the rice and heterozygous *A. thaliana* genomes, suggesting that genome size and complexity may not affect the sequencing depth requirements of these genotypers (Additional file 1: Fig. S13, S14). Besides, genotyping SVs requires more sequencing data than SNPs and indels (Additional file 1: Fig. S12, S14).

### Effects of genome number in the graph

**Fig. 3:**
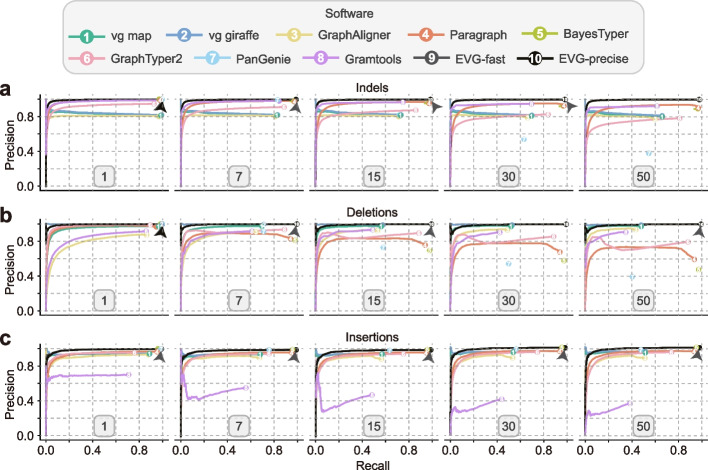
The effect of genome number on the performance of different graph-based genotyping methods. The five ROC curve plots correspond to genotyping results with different numbers (1, 7, 15, 30, 50) of graphed genomes. The genome graph for genotyping is constructed from the *A. thaliana* reference genome and different numbers of alternative genomes. Paired-end (2 × 150 bp) short reads with 30× depth are simulated for genotyping. For each genotyper, precision is plotted against recall as the genotyping quality threshold varies. Read depth on variant sites is used as a substitution score when genotype quality is not available. Arrows indicate the circles hidden by other circles in the plot due to identical or nearly identical precision values. Detailed results are also provided in Additional file 2: Tables S7–S8

As the search space of alignment may expand exponentially when more variants or genomes are graphed, we next evaluated how the number of graphed genomes affects variant genotyping. We reconstructed genome graphs for *A. thaliana* with a range (1, 7, 15, 30, 50) of individual genomes. From our evaluation, only tools Paragraph, BayesTyper, and GraphTyper2 demonstrated relatively stable precision and recall for SNP, indel, and insertion genotyping when graphed genomes increased (Fig. 3a, c; Additional file 1: Fig. S15a). When only seven alternative genomes’ variants were graphed, existing methods could still achieve a good genotyping *F*-score (0.85–0.99 for SNPs, 0.81–0.97 for indels, 0.73–0.93 for deletions, 0.79–0.98 for insertions, 0.01–0.89 for inversions). However, when 50 alternative genomes were incorporated into the genome graph, the *F*-scores of deletion genotyping decreased to only 0.4–0.74. The recall of genotyping for vg map, vg giraffe, and GraphAligner decreased as more genomes were incorporated. For example, as the number of graphed genomes increased from 1 to 50, the recall rate of vg giraffe decreased considerably (0.97 vs. 0.61 for SNPs, 0.99 vs. 0.66 for indels, 0.99 vs. 0.48 for deletions, and 0.92 vs. 0.53 for insertions, 0.55 vs. 0.23 for inversions), while the precision did not change much (Fig. 3a; Additional file 1: Fig. S15). In contrast, Paragraph, BayesTyper, and GraphTyper2 showed greater robustness in terms of recall (Fig. 3a; Additional file 1: Fig. S15). For example, BayesTyper’s recall rates remained above 0.95 for all types of variants except duplication (0.78), but the genotyping precision of deletions and inversions decreased by 0.52 (1.0 vs. 0.48) and 0.49 (1.0 vs. 0.51), respectively. Notably, the insertions precision of PanGenie was higher than that of deletions, possibly because PanGenie needs to count the number of k-mers for each haplotype at nodes. However, as only breakpoint sequences can be used for deletions, this will result in reduced genotyping precision.

### Influence of breakpoint errors on SVs genotyping

**Fig. 4:**
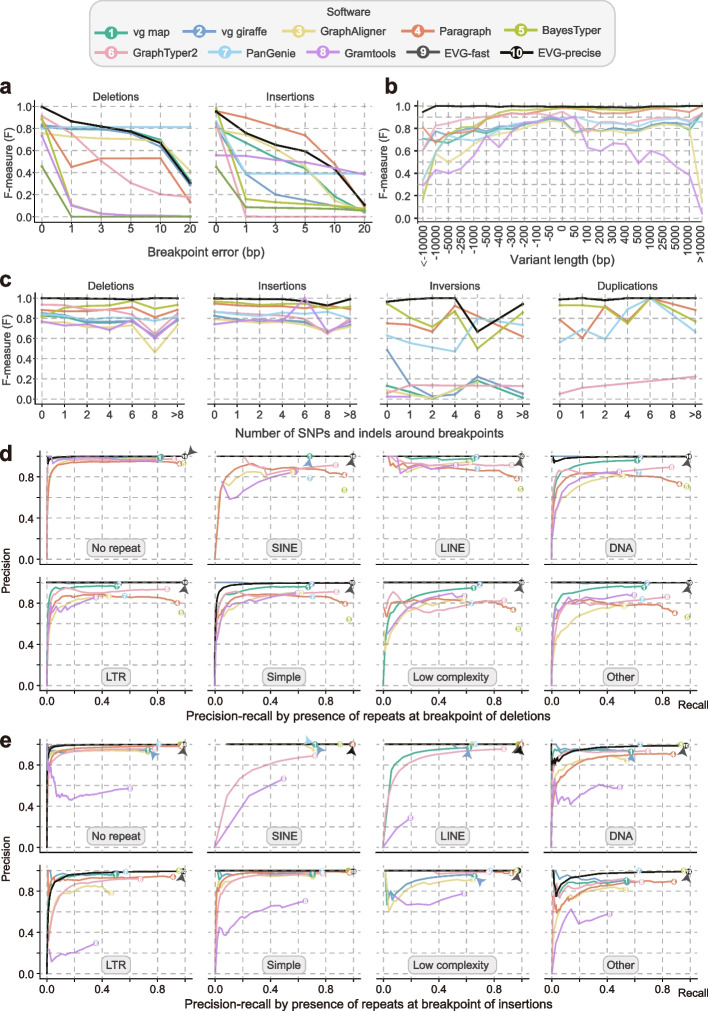
The impact of sequence context and event size on different graph-based genotyping methods. **a** Impact of breakpoint errors on genotyping (0 bp, 1 bp, 3 bp, 5 bp, 10 bp, 20 bp).** b** Impact of variant length (alternative allele length minus reference allele length) on genotyping. **c** Impact of number of SNPs and indels around breakpoints on genotyping. **d**,** e** Effects of repeat sequence types around breakpoints on genotype, partitioned by variant type: **d** deletions and **e** insertions. Different types of repeat sequences are annotated by RepeatMasker. The genome graph for genotyping is constructed from the *A. thaliana* reference genome and seven alternative genomes. Paired-end (2 × 150 bp) short reads with 30× depth are simulated for genotyping. For each genotyper, precision is plotted against recall as the genotyping quality threshold varies. Read depth on variant sites is used as a substitution score when genotype quality is not available. Detailed results are also provided in Additional file 2: Tables S9–S12

The breakpoints of SVs often have some deviation to the true coordinates. To evaluate the tolerance of graph-based genotypers for such SV breakpoint errors, we introduced a 1–20-bp deviation to the true breakpoint coordinates of SVs. For almost all tools, a negative correlation was observed between F-scores and breakpoint deviations (Fig. 4a; Additional file 1: Fig. S16). Consistent with the previous report, BayesTyper, which is based on exact k-mer alignments, was more susceptible to breakpoint deviations. However, another k-mer alignment-based genotyper, PanGenie, performed much better than BayesTyper. This may be because PanGenie can also leverage already known haplotypes to infer genotypes [19].

Overall, the performance on genotyping deletions was better than that of insertions, inversions, and duplications. For genotyping deletions, PanGenie, vg map, vg giraffe, Paragraph, and GraphAligner displayed consistent performance with breakpoint deviations smaller than 10 bp (Fig. 4a). For genotyping insertions, the *F*-scores of vg map, GraphAligner, Paragraph, and Gramtools still had 50% to 90% of that of 0 bp when the breakpoint error reached 5 bp, while the *F*-scores of other software at 1 bp deviation were reduced by more than 50% (Fig. 4a). Further, only Paragraph and vg giraffe maintained performance within a 10 bp error on the genotyping of inversions (Additional file 1: Fig. S16a). However, none of the software was able to tolerate breakpoint errors in duplications (Additional file 1: Fig. S16b).

### Impact of event size and sequence context on SV genotyping

In addition, we stratified SVs based on event size, number of SNPs and indels within breakpoints of 100 bp, and families of repetitive sequences around to estimate the effect of sequence context on SV genotyping. Overall, Paragraph and BayesTyper demonstrated the best genotyping performance across different size ranges of SVs. Although the *F*-scores of both Paragraph and BayesTyper were lower (ranging from 0.17 to 0.81) for deletions larger than 5 kb, they still maintained high *F*-scores (≥ 0.95) for insertions (Fig. 4b). Previous studies have reported that small variants near the breakpoints could affect the accuracy of SV calling [11]. However, our experiment showed that small variants had no serious damage on SV genotyping for these graph-based tools (Fig. 4c), which might be attributed to the improved alignment accuracy as alternative alleles (small variants) are introduced into the graphs.

The genotyping performance of all methods was reduced in the repetitive regions compared to the non-repetitive regions (Fig. 4d, e; Additional file 1: Fig. S7). For example, the GraphAligner *F*-score was even 47% lower in deletions than in non-repetitive regions (Additional file 1: Fig. S7). Compared to other software, Paragraph and BayesTyper demonstrated comparatively stable performance in repetitive regions (Additional file 1: Fig. S7). Repeat sequences had a more severe influence on the genotyping of deletions (17%) than SNPs (9%), indels (11%) and insertions (8%) (Fig. 4d, e; Additional file 1: Fig. S7). Similar to the linear reference-based SV genotyping [11], LTRs had the greatest impact on genotyping. For example, the recalls of deletion genotyping for vg map, vg giraffe, and GraphAligner were reduced by 0.31, 0.31, and 0.36, respectively (Figs. 4d, e).

### Development of an ensemble genotyper

In summary, these graph-based genotypers performed differentially for small and large variants in terms of precision and recall. Further analysis on the overlap of true variant genotyping among the eight genotypers revealed that many variants were not correctly genotyped by some genotypers but were correctly genotyped by others (Additional file 1: Fig. S17, an example shown in Additional file 1: Fig. S18), suggesting an ensemble genotyping strategy may improve genotyping performance.

**Fig. 5:**
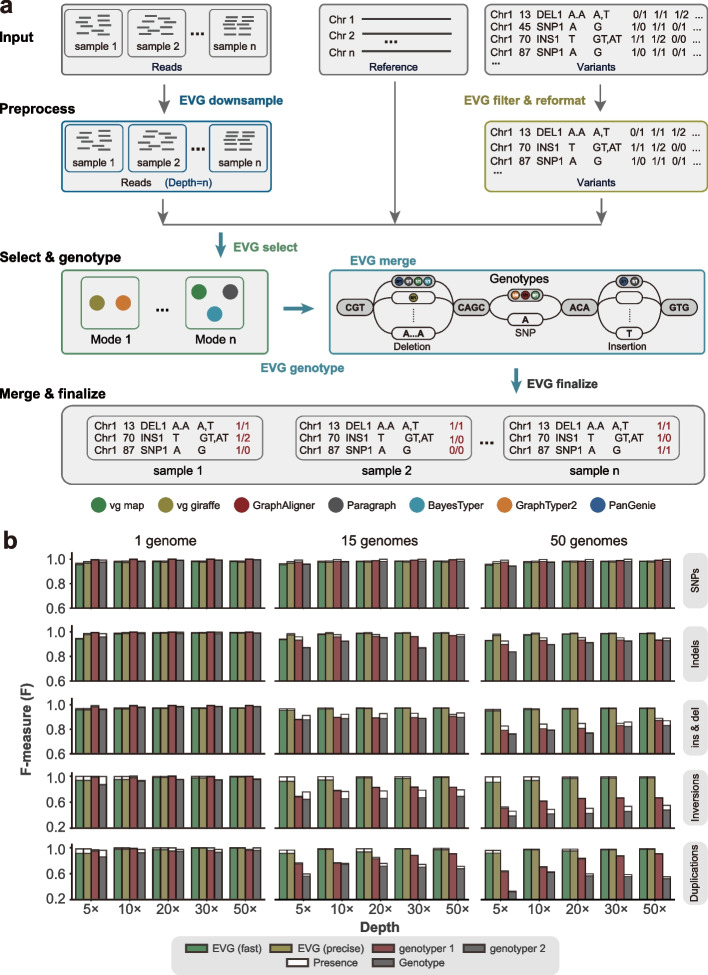
The workflow and performance of the ensemble variant genotyping method EVG.** a** The variant genotyping workflow of EVG mainly consists of three steps: (1) subsample sequencing reads, filter variants, and reformat the input variant VCF file; (2) select one or multiple suitable graph-based genotypers (shown as colored dots) and do genotyping with each of them in parallel; (3) merge the genotype results from step 2 and determine the final genotype for each variant. **b** Genotyping performance of SNPs, indels, ins & del (insertions and deletions), inversions, and duplications on simulated *A. thaliana* genomes under different sequencing depths (5×, 10×, 20×, 30×, 50×) and genome numbers (1, 15, 50). The genome graph for genotyping is constructed from the *A. thaliana* reference genome and different numbers of simulated alternative genomes. Paired-end short-reads (read length: 2 × 150 bp) are simulated for variant genotyping. For each genotyping scenario, the F-measure values of the other two best-performing genotypers are shown here. Transparent and solid bars represent the ability to predict variant “presence” (detection of variant regardless of the genotype) and exact “genotype” (requires both the detection of the variant and agreement between its called genotype and the true genotype). Detailed results are also provided in Additional file 2: Table S13

Here, we developed an Ensemble Variant Genotyper (EVG) by integrating various graph-based genotyping methods (Fig. 5a). Before running the genotyping pipeline, EVG modifies VCF-formatted variants input files provided by users to a common format that can be used for downstream analysis (EVG convert). For instance, graph genotyping tools normally require sequence information from input files but not containing any special characters. Additionally, EVG could filter variants based on minor allele frequency (MAF) and missing rate, if indicated. The EVG pipeline starts with the selection of the most suitable software according to the reference genome size, sequencing data quality, type of variants, and software running requirements (Additional file 1: Fig. S19, see the “Methods” section for details).

To address the issue caused by inconsistent coordinates of the same variants from different software, EVG then clusters the outputs based on the size and position of variants and constructs a variant graph (EVG merge) (Additional file 1: Fig. S20, see the “Methods” section for details). Finally, the most probable combination of genotypes is determined as the genotype with the most support by different genotyping tools at each node (Fig. 5a). To accelerate genotyping, EVG can randomly downsample reads to default depth (15×) when sequencing data is high enough. For oversized genome graphs with numerous variants, EVG offers an optional fast mode where Paragraph is only used for SVs genotyping. These allow EVG to significantly accelerate genotyping while sacrificing very little precision and recall (Fig. 5b).

To assess the performance of EVG, we tested it on all simulated datasets from this study. Firstly, unlike other graph-based genotypers, EVG achieved the highest F-score for both small and large variants with just 5 × 150 bp paired-end short-reads (Fig. 5b; Additional file 1: Fig. S21). Secondly, EVG’s performance was more robust when more genomes were graphed. Specifically, for genome graphs with 50 genomes, EVG achieved a *F*-score above 0.95 for SVs with only 5× short reads, while other best genotypers only reached 0.79 (Fig. 5b; Additional file 1: Fig. S21). In terms of SNP genotyping, the fast mode of EVG performed slightly lower than the best-performing software, as Paragraph is only used for SV genotyping in order to reduce CPU time consumption (Fig. 5b).

### Performance on real data

**Fig. 6:**
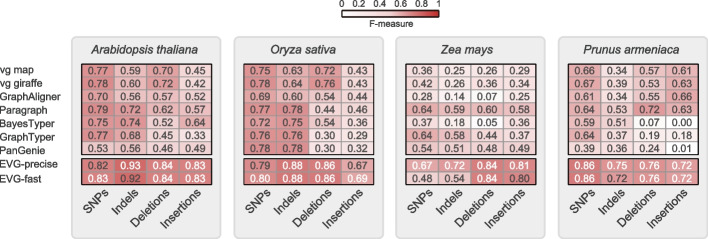
Overall genotyping performance of different graph-based methods based on real short-read data. The read lengths of rice (*Oryza Sativa*), maize (*Zea mays*), and apricot (*Prunus armeniaca*) are all 2 × 150 bp, except for *A. thaliana* (2 × 100 bp). For each plant genome to be genotyped, 30× short reads were used. Each plant graph was constructed from a reference genome and seven alternative genomes

Finally, we carried out testing on real data consisting of 30× Illumina short reads from three diploid homozygous genomes (*A. thaliana*, rice, and maize) [5, 38, 40, 44, 45] as well as one diploid heterozygous genome of Apricot (*Prunus armeniaca*) [46, 47]. Testing was also performed on a genome graph of seven genomes for all genotypers. (Additional file 1: Table S14). Notably, Gramtools was also excluded from the real data testing. The evaluation of maize was also limited to chromosome 10 to reduce resource consumption. In comparison to the simulated data, the *F*-score across genotypers in the real data was lower (Fig. 6). Such a reduction was even worse (< 0.65) for the maize genome, probably because of the high percent of repetitive sequences (Fig. 6; Additional file 1: Fig. S22). For the heterozygous apricot genome, Paragraph had the highest average F-score in all types of variants compared to the other six graph-based genotypers, while BayesTyper was hardly able to genotype the SVs, perhaps due to inaccurate breakpoints (Fig. 6).

We also tested EVG’s performance on all real data sets and found that for all four genomes, EVG achieved the best genotyping performance across all types of variants. Notably, for the largest genome, maize, EVG (precise model) reached the highest *F*-score (0.67 for SNPs, 0.72 for indels, 0.84 for deletions, and 0.81 for insertions) (Fig. 6). Similarly, for the heterozygous genome, EVG showed a comparably higher *F*-score ranging from 0.72 to 0.86 for genotyping small and large variants. More importantly, EVG’s performance in repeated regions was also better than other software, especially for deletion (*F*-score, 0.82) and insertion (*F*-score, 0.83) (Additional file 1: Fig. S22). In summary, our pipeline can be applied to plant genomes with a range of genome sizes or repeat contents compared to the currently available graph-based variant genotypers.

### Runtime and memory usage

The runtime and memory usage of all the methods were measured with the same number of CPUs. As expected, read alignment-based methods such as vg map and Paragraph require relatively more time compared to k-mer alignment-based methods, which were faster despite having larger memory consumption (Additional file 1: Fig. S23a–c, Fig. S24a–c). Additionally, runtime and/or memory increase significantly when graphing more or larger genomes. For instance, the runtime of vg map in maize was more than 14.3 times longer compared to that in *A. thaliana* (Additional file 1: Fig. S23). Compared to other tested tools, GraphTyper2 required the lowest runtime and memory usage for smaller genomes like *A. thaliana* and rice, because it only implemented local alignment in a streamlined sliding window of variants. When tested under the same conditions, EVG’s fast mode required only 6.8 CPU hours in *A. thaliana* and 28.6 CPU hours in rice, and its genotyping was more robust than that of existing methods. The precise mode of EVG further improved the genotyping performance, but it took 27.8 and 118 CPU hours in *A. thaliana* and rice. However, EVG could achieve very close genotyping performance only with 16.7 and 63.8 CPU hours for *A. thaliana* and rice when only 15× short reads were sampled (Additional file 1: Fig. S23, S24).

## Discussion

In this study, we have conducted a comprehensive evaluation of the performance of eight popular graph-based genotypers on multiple representative plant genomes, which are mostly designed and tested only on human genomes. By conducting tests on 25 simulations and 10 real datasets, we have revealed the differences in precision and recall among these tools under different sequencing schemes, genomic context and complexity, and graph size for a spectrum of genetic variants. More importantly, the EVG pipeline developed here can achieve comparably higher genotyping recall and precision even when using 5× short reads and remain stable with an increased number of genomes, fitting the trend requiring population-level millions of variant genotyping.

### Graph-based genotyping variants in plant genomes is challenging

Plant genomes frequently have a large genome size, enriched repetitive sequences, and high sequence diversity or heterozygosity [36, 37]. However, it should be noted that the performance of graph-based genotypers in plants is lower compared to human genomes, particularly when the genome contains high levels of repetitive sequences. The plant genomes have relatively higher repetitiveness (estimated by the percentage of non-unique k-mers of all k-mers in genome) compared to their size-close animal genomes [48]. For example, the 2.3 GB maize genome has 68% such non-unique k-mer compared to that of 18% in human genomes (Additional file 1: Fig. S25a). This poses significant challenges for existing genome graph software, such as those relying on minimizers (derived from k-mers) as seed, leading to imprecise read alignment during the alignment stage. Additionally, tools such as BayesTyper and PanGenie utilize k-mer frequencies for genotyping, but they rely on node-specific k-mers. This may limit the ability to genotype variants that lack enough region-specific k-mers. We also calculated the k-mer frequency at each node of genome graphs for plant and human genomes (Additional file 1: Fig. S25b). It is evident that the proportion of k-mer with node frequency of one is highest in the human genome graph (0.62), while it is lowest in maize (0.17) (Additional file 1: Fig. S25b). This means that if only such specific k-mers are used for genotyping, much few informative k-mers can be utilized in plant genome graphs. Besides, alignment-based software is also affected by repetitive sequences because current graph-based genotyping tools, whether based on the GBWT [33], GCSA2 [34], minimizer (from k-mers) [35], or other indexing algorithms, they followed a similar seed-and-extend alignment strategy. Therefore, the utilization of genome graphs in plants still poses significant challenges, requiring the development of efficient indexing methods tailored for plants.

Additionally, these plant genomic features seriously challenge the efficiency of constructing and indexing pangenome graphs and read alignments. For example, graphing seven maize (only chr10) genomes with vg map requires 90 GB memory (Additional file 1: Fig. S24c). To address these challenges, BayesTyper genotypes local haplotypes by constructing a Bloom filter for selecting which read k-mers should be loaded into memory (i.e., those k-mers stored in the Bloom filter) [18]. However, storing k-mer counts and variant graphs in memory may lead to significant memory consumption, particularly when dealing with large amounts of sequencing data or reference genomes. Alternatively, implementing a Counting Bloom filter or Hierarchical Interleaved Bloom Filter (HIBF) instead of storing k-mer counts in memory may help reduce memory consumption while maintaining the desired level of accuracy [49, 50]. PanGenie, another software program employing k-mer counts, exhibits significant memory consumption despite only considering unique k-mers [19]. While its memory footprint exceeds that of BayesTyper in *A. thaliana*, it is comparatively lower in rice (Additional file 1: Fig. S24a, b). This phenomenon may be attributed to the abundance of repeated k-mers in these plant species that are filtered out during the analysis process.

Unlike k-mer alignment-based genotypers, the vg tool utilizes less memory during genome graph construction and relies on global read alignments [16]. However, the earlier version, vg map requires a significant amount of memory and CPU time for graph indexing, particularly when constructing GCSA2 indexes [34]. To address these issues, vg giraffe [20] (and GraphAligner [28]), use minimizers [35] for seeding to speed up the alignment and reduce memory consumption at the expense of the alignment rate for repeat sequences. In contrast to vg and GraphAligner, GraphTyper2 [17] and Paragraph [24] utilize a local variant realignment strategy based on pre-aligned reads, or solely mapping those around breakpoints. Although such an approach theoretically has the potential to markedly accelerate the genotyping process, our testing revealed that the runtime of Paragraph did not demonstrate significant improvement. This can be attributed primarily to the I/O overhead as three files are generated for each variant during the genotyping. Therefore, despite recent advances in genome graph software, the high variability of plant genomes remains a major challenge for current approaches.

Additionally, SV breakpoints often exhibit errors [10], particularly at repeat-enriched regions, which can affect some genotypers [24], especially those such as BayesTyper, that require long exact matches of k-mers or seeds for read mapping. However, another k-mer-based genotyper, PanGenie, performs much better than BayesTyper by integrating haplotype-resolved pan-genome references. The enriched heterozygous alleles and repetitive sequences will also affect the genotype effect of tools.

Most current graph-based genotypers require 10–20× of data to achieve satisfactory genotyping performance. However, the sequencing depth of early or large genome population sequencing projects in plants is low [39, 51], even only 3–10× for wheat [52]. Future development should consider how to distinguish between sequence errors and real variants especially those in heterozygous loci and repetitive regions with low sequence depth of short reads. One potential approach similar to that implemented in PanGenie is to integrate the known haplotypes of more haplotype-resolved genome assemblies. Additionally, these tools lack stability for population-level pangenome graph-based genotyping. This study found that as the number of variants increased, the performance of some software for certain types of variants continuously decreased while the runtime and memory increased considerably. Taken together, existing methods either require excessive computational resources or sequencing costs or lack stable performance for plant genomes of different complexity, which may limit their applicability in plants.

### Our EVG pipeline helps to do efficient and correct genotyping

It should be noted that these graph-based genotyping tools have complementary advantages (Additional file 1: Fig S17), although they did not exhibit stable and excellent genotyping performance in all testing scenarios. To alleviate the problems of these tools, we have developed the EVG pipeline, which selects the most appropriate process based on reference genome size, sequencing read length, and read depth. EVG pipeline presents more robust genotyping performance compared to existing graph genome methods. More importantly, EVG can reach high *F*-scores of genotyping with only 5× reads, and achieve the peaks with 10× reads, which is normally the average depth of population-level resequencing in plants. Moreover, even with an increased number of nodes in the graph (up to 50 genomes), the genotyping *F*-score of EVG remains above 0.9. (Fig. 5b). Most notably on maize (chr 10), the final genotyping results were better than those obtained using other software, requiring only 52.8 CPU hours (including the time of graph construction, graph indexing, read alignment, and genotyping, 111.3 CPU hours for vg map) (Additional file 1: Fig. S23c). EVG also performs exceptionally well in maize repetitive regions, achieving *F*-scores for deletions and insertions of 0.82 and 0.83, respectively, while the highest values of other software were 0.59 and 0.55 (Additional file 1: Fig. S22).

Although the EVG pipeline has several advantages compared to current graph-based genotypers, it can still be improved in terms of memory consumption and genotyping small variants in the future. The further application of graph-based genotyping in plants requires more consideration for complex regions with dense variants, highly similar regions due to whole genome duplication, and polyploidy genomes. As more high-quality genomes and variants are obtained by long-read sequencing technologies, population-level, and type-full variant genotyping with short reads will be practicable by using graph-based methods, thus facilitating population analysis or trait association studies. By comprehensively testing multiple plant genomes, we reveal the performance level of these graph-based genotypers in different scenarios. Our EVG pipeline with higher performance and stability can be applied to population-scale genotyping for millions of all types of genetic variations for genomes with lower sequencing costs. However, it should be noted that the utilization of genome graphs in plants will require the development of software specifically tailored for plant genomes.

## Conclusions

This paper comprehensively evaluates multiple genome graph-based genotyping software packages using both simulated and real data sets. The results reveal significant challenges in applying existing genome graph software to plants, including resource-intensive computing, poor genotyping accuracy for repeat-related variants, and unstable genotyping performance. The EVG pipeline developed here delivers superior genotyping performance even in repeat regions with minimal increases in resource consumption when only 5× short reads are provided. Our EVG pipeline will be potentially used in population-scale variant genotyping and contribute to plant pan-genomic research.

## Methods

### Selection of variant genotyper

The following graph-based genotypers were selected: vg v1.37.0, GraphAligner v1.0.13, Paragraph v2.3, BayesTyper v1.5, GraphTyper2 v2.7.2, PanGenie v2.0.0, and Gramtools v1.10.0. Both vg map and vg giraffe are used for read alignment in the vg package. The EVG version 1.0.3 was tested in this study. There single reference-based genotyping tools were used including GATK [41] (version 4.2.6.0), DeepVariant [42] (version 1.5.0), and Delly [43] (version 1.1.7).

### Simulated datasets

Overall, the simulated data include sequencing reads from different genomes (*A. thaliana*, rice, maize, *Glycine max, Brassica napus*, heterozygous *A. thaliana*, and heterozygous rice) with different sequencing parameters (technologies, read length, fragment size, and sequencing depth) (Additional file 1: Tables S3, S4). The sequencing reads were used for genotyping variants from public variation databases and/or resulting from genome comparisons.

We used the ART [53] software (version 2.1.8) to generate Illumina paired-end short reads for each of the alternative genomes derived by introducing variants into the reference genomes using the VarSim [54] (version 0.8.6) simulator. For *A. thaliana*, variants from the 1001 Genomes Project and one from our previous study [5, 38] and the reference Col-0 from TAIR10 (https://www.arabidopsis.org) [55] were used. For rice, all types of variations from the Rice SNP-Seek Database (https://snp-seek.irri.org/) [56] and the reference IRGSAP-1.0 (https://rapdb.dna.affrc.go.jp/) [57] were used. For maize, variants are from whole genome comparisons between the reference B73 v5.0 (https://www.maizegdb.org) [40, 45] and previously released assemblies of different accessions [40, 45]. These assemblies were aligned to the reference using Minimap2 [58]. Show-snps from MUMmer4 [59] was used for calling SNPs and indels, and Assemblytics [60] was used for calling SVs. For *Glycine max*, variants from whole genome comparisons between the reference assembly ZH13 (https://download.cncb.ac.cn/gwh/Plants/) [26] and previously released assemblies of different accessions [26]. For *Brassica napus*, variants from whole genome comparisons between the reference assembly ZS11 (http://cbi.hzau.edu.cn/bnapus/) [61] and previously released assemblies of different accessions [61]. The method used for genome comparisons and variant callings was the same as in maize.

The number of introduced variants (Additional file 1: Table S4) is similar to the average number of variants found in real *A. thaliana* accessions [38]. The same control of variant numbers was done for other plant genomes. To evaluate the genotyping when multiple genomes are graphed, we also introduced more variants obtained from the databases described above to simulate multiple genomes (Additional file 1: Table S4).

To evaluate the genotypers’ performance on heterozygous genomes, we simulated heterozygous genomes for *A. thaliana* and rice. Because VarSim cannot specify the degree of heterozygosity, genome heterozygosity can only be controlled by adjusting the number of variants and the percentage of heterozygous variants (with parameters of “vc_prop_het” and “--sv_prop_het”). Finally, five genomes with different heterozygosity rates (0.27%, 0.52%, 1.03%, 2.07%, and 2.35%) were simulated.

To evaluate the genotypers’ performance under different sequencing parameters, we used the ART simulator to simulate short paired-end reads with a range of read length (2×100 bp, 2 × 150 bp, and 2 × 250 bp), fragment size (300 bp, 400 bp, 500 bp, 600 bp) and sequencing depth (5×, 10×, 20×, 30× and 50×). Simulated PacBio sequencing (P6C4 model) was generated using PBSIM [62] (version 2.0.1) with the simulated *A. thaliana* genome serving as the reference. Varying read lengths (20 kb and 75kb bp) and accuracies (0.96 for 20 kb and 0.85 for 75 kb bp) were generated.

### Real datasets

*A. thaliana* real datasets were from the 1001 Genomes Project [5] and one previous study (with NCBI project ID PRJEB31147 [38]), including genome sequences, PacBio, and Illumina sequencing reads (accessions: An-1, C24, and Cvi). Rice real datasets were from the study Zhang et al. [44], downloaded from the CNCB (project ID: PRJCA005926) and TGSrice databases, including genomes, ONT, and Illumina sequencing reads (accessions: TG19, TG28, and TG78). Maize real datasets were from the study Hufford et al [45], downloaded from the ENA (project ID: PRJEB31061) and MaizeGDB databases [40], including genomes, ONT, and Illumina sequencing reads accessions: B97, CML52, and CML69).

To construct the benchmark variant dataset, we first map short reads with bwa [63] (version 0.7.17-r1198-dirty) and call SNPs and indels with GATK [41] (version 4.2.6.0) and BCFtools [64] (version 1.9). We kept variants shared by the two tools, quality score larger than 20 and a sequencing depth lower than 100. For the SV dataset, we mapped long reads using NGMLR [65] (version 0.2.7) with default parameters and subsequently detected SVs using Sniffles [65] (version 2.0.3). In addition, we utilized the nucmer tool [59] (version 4.0.0rc1, parameters: “-c 100,” “-b 500,” and “-l 50”) to align the alternative genome against the reference genome and subsequently identified SVs using Assemblytics [60] (version 1.2.1, parameters: unique sequence length of 10,000, minimum variant size of 50, and maximum variant size of 100,000). We identified those SV common between Sniffles and Assemblytics by filtering those with breakpoint differences of more than 200 bp or event size differences larger than 25% of the real event size. The resulting SVs shared by Sniffles and Assemblytics were used for genotyping.

For genotyping evaluation on heterozygous genomes, we used one haplotype-resolved and chromosome-level assembly of apricot (*P. armeniaca*; cultivar “Rojo Pasión”) from one previous study [46]. The Illumina short reads and PacBio long reads from this study were also used for building the variant dataset. The reference genome from the cultivar “Yinxiangbai” is used for the read mapping [47]. Variants shared by the two haplotypes are homozygous, and the specific ones are heterozygous. To obtain a high-quality variant dataset, we applied both read mapping and assembly comparison-based methods. Firstly, SNPs and indels were called by GATK and BCFtools based on short read mapping, and those common ones were retained, similar to what was described above. Secondly, SVs were called by Sniffles based on the PacBio read alignments resulting from NGMLR. Thirdly, the two haplotype assemblies were aligned to the reference by nucmer (parameters: “-c 100,” “-b 500,” and “-l 50”), followed by calling SNPs and indels with show-snps, and SV with Assemblytics (parameters: unique sequence length of 10,000, minimum variant size of 50, and maximum variant size of 100,000). Finally, variants shared by the read mapping method and the assembly comparison method were identified using the same criteria with other genomes as described above.

### Variant genotyping with simulated and real datasets

For genotyping with simulated homozygous *A. thaliana* and rice genomes, different numbers of genomes (1, 7, 15, 30, and 50) were graphed. Variants across multiple samples were merged to obtain a non-redundant variant input data for downstream analysis of genotyping using VCFtools with the default parameter settings [66]. For genotyping with simulated heterozygous *A. thaliana* and rice, graphs with one and seven genomes were used. For genotyping with simulated maize, *Glycine max* and *Brassica napus*, graphs with one and seven genomes were used. For genotyping with real datasets from *A. thaliana*, rice, maize, and apricot, graphs with one and seven genomes were used. For genotyping evaluation in a real dataset from *A. thaliana*, rice, and maize, variants from three different accessions were genotyped individually. The detailed information on genome graphs and variants is included in Additional file 1: Table S4, S14, S15 and Additional file 2: Table S16, S17.

As genotypers Paragraph, BayesTyper, and GraphTyper2 require linear-reference-based read alignment BAM files, we used BWA to align paired-end short reads from each dataset and used SAMTools [8] (version 1.15) to sort and convert the alignment output into BAM format. vg map, vg giraffe, GraphAligner, PanGenie, and Gramtools all directly input read data, variant data, and reference genomes. For all software except GraphTyper2, we run the genotyping with the default parameters as recommended in their manuals. The detailed commands for running each genotyping tool on a Linux system are uploaded to the GitHub website (https://github.com/JiaoLab2021/EVG/wiki/EVG-paper).

To measure the genotyping precision, recall, and F-score, we compared each variant called by each genotyper with the true variant dataset by using the script graphvcf in EVG developed in this study. For SVs, if both the start breakpoint and the end breakpoint of one SV were within 200 bp of the true SV positions, and the SV sizes differed by at most 25% of the true size, such SV calling was considered correct presence. If the genotype of this SV calling also matched the true event, it is considered correct genotype. For an indel calling, the correct presence requires a position difference of less than 10 bp, while for a SNP calling, an exact position match is necessary.

### The EVG workflow

EVG software takes as input a variant VCF file of the population, the reference genome, and a configuration file containing the sequencing reads path. The whole EVG workflow mainly contains three steps. EVG can support restarting the task from the point of failure by using the “--restart” parameter. The details of each step are described as follows:

#### Step 1: preprocessing

This preprocessing step mainly involves the read data subsampling, variant filtering, and VCF file reformatting. Firstly, to speed up read mapping against genome graphs, EVG offers a solution by first extracting a subset (default: 15×) of read data for downstream genotyping. Based on this study, acceptable genotyping results can be achieved with as little as 5× data. Secondly, to reduce resource consumption, variants can be filtered according to their Minor Allele Frequency (MAF) and missing rate using EVG. By default, variants are not filtered. Thirdly, to avoid throwing errors due to incompatible input variants in the VCF file provided by users, EVG automatically checks the VCF file and reformats any variants to be compatible with the software’s different requirements accordingly.

#### Step 2: select genotypers and do genotyping

EVG automatically selects the optimal genotyping process based on factors including the size of the reference genome, the sequencing depth of the individual genome to be genotyped, and the read length of the sequencing data. In particular, if long-read data are used for genotyping, EVG will run GraphAligner for aligning reads to the graph and vg for the downstream genotyping. For short reads-based genotyping, two modes EVG-fast and EVG-precise, are provided. BayesTyper is used for SNP, indels, and SV genotyping in both modes. In the EVG-fast mode, the tool Paragraph is only used for SV genotyping, and other tools are selected for SNP, indel, and SV genotyping according to the reference genome size, sequencing read length and depth. Specifically, if the genome size is larger than 1 GB, EVG will include vg giraffe; otherwise, vg map will be added. Additionally, EVG will also run GraphTyper2 when the read length is greater than 130 bp and the sequencing depth is greater than 5×. However, when the sequencing depth is less than 5× or read length is shorter than 130bp, EVG will employ PanGenie as well (Additional file 1: Fig. S19; Additional file 2: Table S18). In the EVG-precise mode, Paragraph is used for SNP, indels, and SV genotyping. Like in EVG-fast mode, other tools are selected for SNP, indel, and SV genotyping according to the reference genome size, sequencing read length and depth. Notably, EVG offers the option to customize the software for genotyping.

Remarkably, Paragraph generates three files for each variant during running, which will result in high disk I/O consumption if SNPs and indels are included. So, Paragraph is only used for SV genotyping in EVG-fast mode. After selecting, EVG submits all tasks in parallel. When all tasks are completed, EVG converts the output for subsequent merges.

#### Step 3: merge and finalize genotyping

For each sample, the input SVs to be genotyped are the same. But some graph-based programs may output different coordinates for some SVs. For example, variant coordinates obtained from vg may have some changes. Here, our *EVG merge* process aims to determine the true coordinates of the output SVs which they should correspond to. EVG employs a clustering approach based on variant size and position to cluster the output (*EVG merge*) (Additional file 1: Fig. S20). For variants larger than 50 bp, EVG clusters them together if their positional differences are less than 200 bp. Similarly, for indels smaller than 50 bp, their positional differences should be less than 10 bp, while SNPs require precise positional matching. In addition to considering positional information, EVG also require that the length difference ratio of variants within the same cluster is less than 0.25.

EVG will first cluster all the variants to form a variant graph, with each node containing the variant’s location, length, variant type, genotyping, and depth information. The variants in the same position are put on the same branch. To keep the read depth of different software on the same order of magnitude, we use the *Z*-score to normalize the read depth for each variant:

$$D{\prime}=\frac{D-\mu }{\sigma }$$

$$\sigma =\sqrt{\frac{\sum_{i=1}^{N}{\left({D}_{i}-\mu \right)}^{2}}{N}}$$

where *D'* indicates the normalized read depth of a variant. *D* corresponds to this variant read depth calculated by genotypers and present in the genotyping output VCF file. *μ* and $\sigma$ are the average and standard deviation of read depth for all variants genotyped by a genotyper. *N* is the number of variants.

EVG selects the most likely genotyping according to consistency across different genotypers and depth information. For small variants, including SNPs and indels, the genotype is determined as the one supported by most genotypers. When no genotype is supported by more than one genotyper, EVG will skip this variant. For SV genotyping, EVG also chose the genotype supported by most genotypers. In cases where all genotype frequencies are equal to 1, the genotype is the one with the smallest normalized absolute depth.

### Supplementary Information

**Additional file 1. Supplementary figures and tables: Supplementary figures S1-S25, and Supplementary tables S1-S4 and S14-S15.**

**Additional file 2. Supplementary tables: Supplementary tables S5-S13 and S16-S18.**

**Additional file 3.** Review history.

## Data Availability

The *Arabidopsis thaliana data* was obtained from the 1001 Genomes Project [5] and our previously published 7 *A. thaliana* genomes (An-1, C24, Cvi-0, Eri-1, Kyo, Ler, Sha) [38]. Re-sequencing and PacBio data were downloaded from the European Nucleotide Archive database (project ID: PRJEB31147) [38]. For the rice variation data, it was sourced from the Rice SNP-Seek Database [56], and the genome data was obtained from the TGSrice database [44], while the re-sequencing and ONT data were downloaded from the China National Center for Bioinformation database (project ID: PRJCA005926) [44]. The maize data was obtained from the MaizeGDB database [40], and the re-sequencing data was downloaded from the European Nucleotide Archive database (project ID: PRJEB31061) [45]. For the soybean population genome data, it was sourced from the China National Center for Bioinformation database (project ID: PRJCA002030) [26]. The *Brassica napus* genome data was obtained from the BnPIR database [61]. The apricot genome data was downloaded from the European Nucleotide Archive database (project ID: PRJEB37669) [46], and the reference apricot genome was downloaded from NCBI (project ID: PRJNA577047) [47]. The re-sequencing data was downloaded from the European Nucleotide Archive (project ID: PRJEB37669) [46] and CNGBdb (project ID: CNP0000718) [47]. The source code of EVG is publicly available under MIT license on GitHub [67] and Zenodo [68].

## References

[1] Ho SS, Urban AE, Mills RE (2020). Structural variation in the sequencing era. Nat Rev Genet.

[2] Alkan C, Coe BP, Eichler EE (2011). Genome structural variation discovery and genotyping. Nat Rev Genet.

[3] Fuentes RR, Chebotarov D, Duitama J, Smith S, De la Hoz JF, Mohiyuddin M, Wing RA, McNally KL, Tatarinova T, Grigoriev A (2019). Structural variants in 3000 rice genomes. Genome Res.

[4] Wang W, Mauleon R, Hu Z, Chebotarov D, Tai S, Wu Z, Li M, Zheng T, Fuentes RR, Zhang F (2018). Genomic variation in 3,010 diverse accessions of Asian cultivated rice. Nature.

[5] Genomes Consortium (2016). Electronic address mngoaa, Genomes C: 1,135 Genomes Reveal the Global Pattern of Polymorphism in Arabidopsis thaliana. Cell.

[6] Jiao WB, Patel V, Klasen J, Liu F, Pecinkova P, Ferrand M, Gy I, Camilleri C, Effgen S, Koornneef M (2021). The Evolutionary Dynamics of Genetic Incompatibilities Introduced by Duplicated Genes in Arabidopsis thaliana. Mol Biol Evol.

[7] Mahmoud M, Gobet N, Cruz-Davalos DI, Mounier N, Dessimoz C, Sedlazeck FJ (2019). Structural variant calling: the long and the short of it. Genome Biol.

[8] Danecek P, Bonfield JK, Liddle J, Marshall J, Ohan V, Pollard MO, Whitwham A, Keane T, McCarthy SA, Davies RM, Li H: Twelve years of SAMtools and BCFtools. Gigascience. 2021; 10:giab008. 10.1093/gigascience/giab008 PMC7931819 33590861

[9] DePristo MA, Banks E, Poplin R, Garimella KV, Maguire JR, Hartl C, Philippakis AA, del Angel G, Rivas MA, Hanna M (2011). A framework for variation discovery and genotyping using next-generation DNA sequencing data. Nat Genet.

[10] Kosugi S, Momozawa Y, Liu X, Terao C, Kubo M, Kamatani Y (2019). Comprehensive evaluation of structural variation detection algorithms for whole genome sequencing. Genome Biol.

[11] Cameron DL, Di Stefano L, Papenfuss AT (2019). Comprehensive evaluation and characterisation of short read general-purpose structural variant calling software. Nat Commun.

[12] Ballouz S, Dobin A, Gillis JA (2019). Is it time to change the reference genome?. Genome Biol.

[13] Sherman RM, Salzberg SL (2020). Pan-genomics in the human genome era. Nat Rev Genet.

[14] Computational Pan-Genomics C (2018). Computational pan-genomics: status, promises and challenges. Brief Bioinform.

[15] Eizenga JM, Novak AM, Sibbesen JA, Heumos S, Ghaffaari A, Hickey G, Chang X, Seaman JD, Rounthwaite R, Ebler J (2020). Pangenome Graphs. Annu Rev Genomics Hum Genet.

[16] Hickey G, Heller D, Monlong J, Sibbesen JA, Siren J, Eizenga J, Dawson ET, Garrison E, Novak AM, Paten B (2020). Genotyping structural variants in pangenome graphs using the vg toolkit. Genome Biol.

[17] Eggertsson HP, Kristmundsdottir S, Beyter D, Jonsson H, Skuladottir A, Hardarson MT, Gudbjartsson DF, Stefansson K, Halldorsson BV, Melsted P (2019). GraphTyper2 enables population-scale genotyping of structural variation using pangenome graphs. Nat Commun.

[18] Sibbesen JA, Maretty L, Danish Pan-Genome C, Krogh A (2018). Accurate genotyping across variant classes and lengths using variant graphs. Nat Genet.

[19] Ebler J, Ebert P, Clarke WE, Rausch T, Audano PA, Houwaart T, Mao Y, Korbel JO, Eichler EE, Zody MC (2022). Pangenome-based genome inference allows efficient and accurate genotyping across a wide spectrum of variant classes. Nat Genet.

[20] Siren J, Monlong J, Chang X, Novak AM, Eizenga JM, Markello C, Sibbesen JA, Hickey G, Chang PC, Carroll A (2021). Pangenomics enables genotyping of known structural variants in 5202 diverse genomes. Science.

[21] Garrison E, Sirén J, Novak AM, Hickey G, Eizenga JM, Dawson ET, Jones W, Garg S, Markello C, Lin MF (2018). Variation graph toolkit improves read mapping by representing genetic variation in the reference. Nat Biotechnol.

[22] Eggertsson HP, Jonsson H, Kristmundsdottir S, Hjartarson E, Kehr B, Masson G, Zink F, Hjorleifsson KE, Jonasdottir A, Jonasdottir A (2017). Graphtyper enables population-scale genotyping using pangenome graphs. Nat Genet.

[23] Rakocevic G, Semenyuk V, Lee WP, Spencer J, Browning J, Johnson IJ, Arsenijevic V, Nadj J, Ghose K, Suciu MC (2019). Fast and accurate genomic analyses using genome graphs. Nat Genet.

[24] Chen S, Krusche P, Dolzhenko E, Sherman RM, Petrovski R, Schlesinger F, Kirsche M, Bentley DR, Schatz MC, Sedlazeck FJ, Eberle MA (2019). Paragraph: a graph-based structural variant genotyper for short-read sequence data. Genome Biol.

[25] Qin P, Lu H, Du H, Wang H, Chen W, Chen Z, He Q, Ou S, Zhang H, Li X (2021). Pan-genome analysis of 33 genetically diverse rice accessions reveals hidden genomic variations. Cell.

[26] Liu Y, Du H, Li P, Shen Y, Peng H, Liu S, Zhou GA, Zhang H, Liu Z, Shi M (2020). Pan-Genome of Wild and Cultivated Soybeans. Cell.

[27] Zhou Y, Zhang Z, Bao Z, Li H, Lyu Y, Zan Y, Wu Y, Cheng L, Fang Y, Wu K (2022). Graph pangenome captures missing heritability and empowers tomato breeding. Nature.

[28] Rautiainen M, Marschall T (2020). GraphAligner: rapid and versatile sequence-to-graph alignment. Genome Biol.

[29] Letcher B, Hunt M, Iqbal Z (2021). Gramtools enables multiscale variation analysis with genome graphs. Genome Biol.

[30] Kim D, Paggi JM, Park C, Bennett C, Salzberg SL (2019). Graph-based genome alignment and genotyping with HISAT2 and HISAT-genotype. Nat Biotechnol.

[31] Hunt M, Letcher B, Malone KM, Nguyen G, Hall MB, Colquhoun RM, Lima L, Schatz MC, Ramakrishnan S (2022). consortium CR, Iqbal Z: Minos: variant adjudication and joint genotyping of cohorts of bacterial genomes. Genome Biol.

[32] Grytten I, Dagestad Rand K, Sandve GK (2022). KAGE: fast alignment-free graph-based genotyping of SNPs and short indels. Genome Biol.

[33] Siren J, Garrison E, Novak AM, Paten B, Durbin R (2020). Haplotype-aware graph indexes. Bioinformatics.

[34] Sirén J: Indexing Variation Graphs. 19th Workshop on Algorithm Engineering and Experiments (ALENEX) 2017, SIAM, 2017:13-27.

[35] Roberts M, Hayes W, Hunt BR, Mount SM, Yorke JA (2004). Reducing storage requirements for biological sequence comparison. Bioinformatics.

[36] Sun Y, Shang L, Zhu QH, Fan L, Guo L (2022). Twenty years of plant genome sequencing: achievements and challenges. Trends Plant Sci.

[37] Marks RA, Hotaling S, Frandsen PB, VanBuren R (2021). Representation and participation across 20 years of plant genome sequencing. Nat Plants.

[38] Jiao WB, Schneeberger K (2020). Chromosome-level assemblies of multiple Arabidopsis genomes reveal hotspots of rearrangements with altered evolutionary dynamics. Nat Commun.

[39] The 3,000 rice genomes project. The 3,000 rice genomes project. GigaScience. 2014;3:2047-2217X-2043-2047.

[40] Woodhouse MR, Cannon EK, Portwood JL, Harper LC, Gardiner JM, Schaeffer ML, Andorf CM (2021). A pan-genomic approach to genome databases using maize as a model system. BMC Plant Biol.

[41] McKenna A, Hanna M, Banks E, Sivachenko A, Cibulskis K, Kernytsky A, Garimella K, Altshuler D, Gabriel S, Daly M, DePristo MA (2010). The Genome Analysis Toolkit: a MapReduce framework for analyzing next-generation DNA sequencing data. Genome Res.

[42] Poplin R, Chang P-C, Alexander D, Schwartz S, Colthurst T, Ku A, Newburger D, Dijamco J, Nguyen N, Afshar PT (2018). A universal SNP and small-indel variant caller using deep neural networks. Nat Biotechnol.

[43] Rausch T, Zichner T, Schlattl A, Stutz AM, Benes V, Korbel JO (2012). DELLY: structural variant discovery by integrated paired-end and split-read analysis. Bioinformatics.

[44] Zhang F, Xue H, Dong X, Li M, Zheng X, Li Z, Xu J, Wang W, Wei C (2022). Long-read sequencing of 111 rice genomes reveals significantly larger pan-genomes. Genome Res.

[45] Hufford MB, Seetharam AS, Woodhouse MR, Chougule KM, Ou SJ, Liu JN, Ricci WA, Guo TT, Olson A, Qiu YJ (2021). De novo assembly, annotation, and comparative analysis of 26 diverse maize genomes. Science.

[46] Campoy JA, Sun H, Goel M, Jiao WB, Folz-Donahue K, Wang N, Rubio M, Liu C, Kukat C, Ruiz D (2020). Gamete binning: chromosome-level and haplotype-resolved genome assembly enabled by high-throughput single-cell sequencing of gamete genomes. Genome Biol.

[47] Zhang QP, Zhang DY, Yu K, Ji JJ, Liu N, Zhang YP, Xu M, Zhang YJ, Ma XX, Liu S (2021). Frequent germplasm exchanges drive the high genetic diversity of Chinese-cultivated common apricot germplasm. Hortic Res.

[48] Jiao WB, Schneeberger K (2017). The impact of third generation genomic technologies on plant genome assembly. Curr Opin Plant Biol.

[49] Fan L, Cao P, Almeida J, Broder AZ (2000). Summary cache: a scalable wide-area Web cache sharing protocol. IEEE/ACM Trans Netw.

[50] Mehringer S, Seiler E, Droop F, Darvish M, Rahn R, Vingron M, Reinert K (2023). Hierarchical Interleaved Bloom Filter: enabling ultrafast, approximate sequence queries. Genome Biol.

[51] Mills RE, Walter K, Stewart C, Handsaker RE, Chen K, Alkan C, Abyzov A, Yoon SC, Ye K, Cheetham RK (2011). Mapping copy number variation by population-scale genome sequencing. Nature.

[52] Zhao X, Guo Y, Kang L, Yin C, Bi A, Xu D, Zhang Z, Zhang J, Yang X, Xu J (2023). Population genomics unravels the Holocene history of bread wheat and its relatives. Nat Plants.

[53] Huang W, Li L, Myers JR, Marth GT (2012). ART: a next-generation sequencing read simulator. Bioinformatics.

[54] Mu JC, Mohiyuddin M, Li J, Bani Asadi N, Gerstein MB, Abyzov A, Wong WH, Lam HY (2015). VarSim: a high-fidelity simulation and validation framework for high-throughput genome sequencing with cancer applications. Bioinformatics.

[55] Lamesch P, Berardini TZ, Li D, Swarbreck D, Wilks C, Sasidharan R, Muller R, Dreher K, Alexander DL, Garcia-Hernandez M (2012). The Arabidopsis Information Resource (TAIR): improved gene annotation and new tools. Nucleic Acids Res.

[56] Mansueto L, Fuentes RR, Borja FN, Detras J, Abriol-Santos JM, Chebotarov D, Sanciangco M, Palis K, Copetti D, Poliakov A (2017). Rice SNP-seek database update: new SNPs, indels, and queries. Nucleic Acids Res.

[57] Kawahara Y, de la Bastide M, Hamilton JP, Kanamori H, McCombie WR, Ouyang S, Schwartz DC, Tanaka T, Wu J, Zhou S, et al: Improvement of the Oryza sativa Nipponbare reference genome using next generation sequence and optical map data. Rice. 2013; 6:4. 10.1186/1939-8433-6-4 PMC5395016 24280374

[58] Li H (2018). Minimap2: pairwise alignment for nucleotide sequences. Bioinformatics.

[59] Marcais G, Delcher AL, Phillippy AM, Coston R, Salzberg SL, Zimin A (2018). MUMmer4: A fast and versatile genome alignment system. PLoS Comput Biol.

[60] Nattestad M, Schatz MC (2016). Assemblytics: a web analytics tool for the detection of variants from an assembly. Bioinformatics.

[61] Song JM, Guan Z, Hu J, Guo C, Yang Z, Wang S, Liu D, Wang B, Lu S, Zhou R (2020). Eight high-quality genomes reveal pan-genome architecture and ecotype differentiation of Brassica napus. Nat Plants.

[62] Ono Y, Asai K, Hamada M (2013). PBSIM: PacBio reads simulator–toward accurate genome assembly. Bioinformatics.

[63] Li H: Aligning sequence reads, clone sequences and assembly contigs with BWA-MEM. arXiv. 2013:13033997v2.

[64] Li H (2011). A statistical framework for SNP calling, mutation discovery, association mapping and population genetical parameter estimation from sequencing data. Bioinformatics.

[65] Sedlazeck FJ, Rescheneder P, Smolka M, Fang H, Nattestad M, von Haeseler A, Schatz MC (2018). Accurate detection of complex structural variations using single-molecule sequencing. Nat Methods.

[66] Danecek P, Auton A, Abecasis G, Albers CA, Banks E, DePristo MA, Handsaker RE, Lunter G, Marth GT, Sherry ST (2011). The variant call format and VCFtools. Bioinformatics.

[67] Du ZZ, He JB, Jiao WB. A comprehensive benchmark of graph-based genetic variant genotyping algorithms on plant genomes for creating an accurate ensemble pipeline. GitHub 2023. https://github.com/JiaoLab2021/EVG. 10.1186/s13059-024-03239-1 PMC11003132 38589937

[68] Du ZZ, He JB, Jiao WB. A comprehensive benchmark of graph-based genetic variant genotyping algorithms on plant genomes for creating an accurate ensemble pipeline. 2024. Zenodo. 10.5281/zenodo.10791273. 10.1186/s13059-024-03239-1 PMC11003132 38589937

