# Supplementary material for: A comprehensive benchmark of graph-based genetic variant genotyping algorithms on plant genomes for creating an accurate ensemble pipeline
Source: Genome Biol. 2024 Apr 8;25:91. doi: 10.1186/s13059-024-03239-1 (PMC11003132; doi:10.1186/s13059-024-03239-1)
Supplement: Supplementary file 3 — Additional file 3. Review history. [file 13059_2024_3239_MOESM3_ESM.docx]

**1^st^ round**

**Reviewer 1**

Focusing on the genotyping issue in plants, the authors conducted a comprehensive benchmarking study to evaluate the performance of eight selected genotyping software, taking into account various factors that can influence genotyping outcomes in plants. Based on the benchmarking results, the authors identified the most suitable software for different scenarios, which were then integrated into a package with an internal pipeline. This package enables researchers to genotype all types of variants in any plant species, with reduced requirements for computational resources and sequencing data. The authors effectively addressed the significant and practical topic of modern genotyping in plants by evaluating and integrating existing tools. Their thorough understanding of the issue is evident through their in-depth investigation of relevant tools, algorithms, and other aspects. The benchmarking work is well-designed, supported by authentic data, and the developed package meets expectations as a product. The manuscript is enriched with informative figures and tables. In summary, this manuscript represents valuable and excellent work. However, there are still some issues in the manuscript that need to be addressed and improved before its publication.
1. The main result claimed in title is "a comprehensive benchmark of … algorithms", but the benchmarking is actually done on several tools with overlapped algorithms, and less algorithm anatomy/analyze is given through the manuscript. I suggest the author to enhance the description of the innovative algorithm in the EVG.
2. I didn't see how variant with differed coordinates from each genotypers are merged. This is critical to the pipeline.
3. The genome size of Brassica napus is approximately 1Gb. However, it is an allopolyploid. Its heterozygosity differs significantly from several other species. In order to achieve a more comprehensive range of genome size, I suggest incorporating another diploid species with a genome size of 1G.
4. (line 66) The vg toolkit developed by Erik Garrison's team is officially denoted in all lowercase (together with its subprograms). Better to mention it as "vg map" "vg giraffe" without hyphen. (All upper case "VG" exists some places in the docs, exhibiting little inconsistency. But largely and especially in publication it's presented as "vg".)
5. (line 180) Read depth is an indicator other than genotype quality, thus the relation should be "substitution when lack" rather than "proxy". Moreover, DP and GQ are not presented in the figure, so abbreviation should belong to elsewhere in the writing (line 134 as their first presence).
6. (line 264) "breakpoint deviations" is mentioned several times in this section, but a concise definition is lacking.
7. (line 284) The variant length is clarified with a formula in parenthesis, however it may become misleading. In fact, I took some while to confirm that it is a minus expression rather than two phrase connected with hyphen.
8. (line 340-341) "when sequencing data is high enough". How much coverage is enough?
9. The legend of Figure 5b may be confusing, especially the term "presence". Presence variation?
10. To achieve consistent result with the author have tested through, the version and parameters of the involved tools should be locked and adhere to versioning of the main program EVG (source code).

**Reviewer 2**

The paper consists of two parts (1) a comprehensive evaluation of eight graph-based genotypes, and the introduction on an "ensemble" genotyper EVG that uses information from all either genotypers to decide the best answer for each variant. This is shown to have overall better F-measure than all eight ones individually.

Overall, I find the paper interesting: via its first part it helps biologists understand the performance of state-of-the-art genotypes, and via its second part it proposes a method to get the best answer from all, for overall better accuracy, and which works even with 5x coverage.

However, I have some questions and comments that would need to be answered before confirming the above conclusions. My major comment is that I did not find a strong motivation on why we should be using a graph-based genotyper in the first place for complex plant genomes. For example, if I understood correctly, the ground-truth for benchmarking the real dataset was constructed using single reference(s), which to me it implies that the paper trusts more genotyping using single reference(s) than graph-based ones.

Specific comments:

As far as I understand, the conclusion of section "Effects of genome number in the graph" is that the more genomes in the graph, the worse the results. While I agree that the larger this number, the more complex the graphs become, and the harder the alignment problem can become, this appears to me as counter-intuitive to the whole reason of building a pan-genome graphs and calling variants there. If alignment gets worse, then why work with genome graphs for plant genomes in the first place?

Can we conclude that genome graphs are a helpful tool for genomes such as human, and not for plant genomes? If so, I find this to be a major drawback for this work, because then one should just to variant calling using single reference(s).

Following up on the above point, I think you should also include in your evaluation (both in part (1) and part (2)) a couple of state-of-the-art variant callers working with only reference genomes (not graphs). This would be the baseline, and this would motivate using genome graphs in the first place for such complex genomes.

I did not find how from some graph aligners (e.g. Graph Aligner) you call variants. I quickly checked the GraphAligner manual (https://github.com/maickrau/GraphAligner) and I did not find some option that it would support this. If you use an external tool, could it introduce some bias?

I do not understand how the plots in Figs 1, 2, ... are made. You say "For each genotyper, precision is plotted against recall as the genotyping quality threshold varies." Can you explain more precisely?

I also have a major comment about the way the experimental results in both parts of the paper are presented. In part (1) you express these as ROC curve where precision and recall are directly visible. In part (2) you combine these into F-measure, but potential users cannot tell e.g. how precise EVG is. I think there should be a uniform evaluation.

Following up on the above, in part (1) you have extensive experimental scenarios, but in part (2) you are not very extensive in experimenting with your tool EVG. I find this to be a major issue: if you have a comprehensive evaluation of eight genotypers, why not test your novel genotyper in the same comprehensive evaluation? A way to address this could be to describe EVG before the evaluation, and include EVG in all scenarios of the comprehensive evaluation.

In part (2), could you give more technical details on the methods of EVG? For example, section "Step 3: merge and finalize genotyping" could be enhanced with one or two figures and some more explanations.

Could you give a more intuitive explanation of how come your tool is overall better than all other genotypes, by not using any novel insights? Is it that it takes the "best" answer for each variant, and thus it is overall better?

In Sec "Performance on real data", you construct a ground-truth for this real dataset by aligning the reads to a single reference (if I understand correctly). But then again this defeats the message of the paper: if you consider single-reference alignments as ground-truth, then why should we do graph-based genotyping in the first place?

Even more critical, if in part (1) you show that graph-based genotypers are more accurate than ones based on single reference(s), then why should we construct the ground-truth for real data using genoetypers based on single reference(s)?

**Reviewer 3**

The authors provide a comprehensive benchmark and comparison of genome-graph-based genotypers on plant genomes. Given that most of these methods were originally developed for the human genome, their performance on plant genomes remains unclear. The benchmark and comparison presented in this manuscript offer useful insights.

Additionally, the authors propose an ensemble-based genotype calling approach by combining different genotypers and demonstrate, through simulation data, that the ensemble approach performs better. I have the following comments on the experiment design and results, for which I hope the authors can provide further data support.

Major:
1. One major assumption made in this study is that graph-genome-based genotyping outperforms linear reference-based approaches. However, it appears that the performance in SNP and INDEL genotype calling is less favorable compared to earlier results reported by GATK and DeepVariant. I wonder whether this discrepancy is due to the nature of the data being sourced from plant genomes or if it indicates a general limitation of the graph-genome approach in SNP/INDEL genotyping. To address this, it would be beneficial if the authors could provide additional supporting results and analyses.

2. The evaluation of the proposed ensemble approach, EVG, is exclusively benchmarked on simulation data. I wonder how well the performance of the EVG approach when applied to real data from the human genome. There are high-quality benchmark datasets available from the Human Pan-Genome Project (https://www.nature.com/articles/s41586-023-05896-x#data-availability), which includes T2T haplotype-resolved assemblies and 30X whole-genome sequencing (WGS) short reads that are publicly available. Incorporating such real-world data into the evaluation could provide valuable insights into the practical utility of the EVG method.

3. The selected variations for the simulation dataset have been curated from cohort-released results primarily based on short reads. It is noteworthy that a substantial number of variations are not identified when compared to results obtained from long reads. For instance, in the case of polymorphic transposons within the reference genome, most long-read callers can accurately identify them as deletions, whereas short-read callers often miss or misidentify them. Despite the inherent challenges in identifying such variations from short reads, it would be beneficial to assess the performance of graph-based genotypers in accurately genotyping these instances. This additional evaluation would provide valuable insights into the effectiveness of graph-based methods in handling challenging scenarios like polymorphic transposons in short-read data..


Minor:
1. The colors in the Figures are close to each other. The authors should change to more distinguishable colors to enhance clarity and readability.
2. The mutations used for generating the simulation data, as well as the commands for running each tool, should be provided to facilitate the reproducibility of the results.

**Reviewer 4**

Du et al. describe the benchmark analysis of a set of graph-based tools in genotyping genetic variants in plant genomes. Based on their analysis results, the authors developed an Ensemble Variant Genotyper (EVG) that can integrate variants genotyped with different existing graph-based genotypers, each of which displayed advantages in genotyping certain categories of variants in plant genomes of different sizes and complexities.

When genotyping variants using a graph-based pan-genome, the samples are normally not those that are used in the pan-genome graph construction. Therefore, the authors should conduct the "leave-one-out" experiment to evaluate the performance of different genotypers, as described in the PanGenie paper (https://www.nature.com/articles/s41588-022-01043-w). The current approach used by the authors for evaluation could be biased.

In the abstract (Line 37), the authors claimed that EVG has a better genotyping performance while "without increasing resource consumption". This seems not true. Based on what the authors stated in the manuscript, in most cases at least two genotypers will be run in EVG (Line 607-615). In the software homepage (https://github.com/JiaoLab2021/EVG), it says that "In fast mode, only certain software is utilized to genotype SNPs and indels, while precise mode employs all software to genotype all variants". This will definitely and substantially increase computational resource consumption (especially for the precise mode). In fact, Fig. S19 clearly shows that EVG consumed more CPUs than most of the other genotypers. Nonetheless, it is not clear how EVG can achieve less computational resource consumption as it employs all software to genotype all variants, e.g., Paragraph was at least included in EVG for the variant genotyping (Line 609-611), then how could Paragraph consume more CPU than EVG in all cases (Fig. S19)? Could this be due to the downsampling of the input data in EVG? If this is the case, then the comparison is biased.

The author concluded that including more genomes in the graph would decrease the performances of all genotypers (Line 231-263). This is counterintuitive as more genomes included in the graph will improve the read mapping and therefore improve the variant genotyping (this is why more and more studies are focusing on graph-based pan-genomes constructed from multiple genomes). This could indicate that the variants and/or the simulated reads used for evaluation might not be representative.

While I understand the complexity of the human genome is different from that of plant genomes, I am very surprised to see that the performances of the genotypers in all four plant species evaluated here (Fig. 6) are substantially worse than those reported in human (Fig. 3 of the PanGenie paper; https://www.nature.com/articles/s41588-022-01043-w). Actually, Arabidopsis genome is much simpler than the human genome, therefore a better or comparable performance should be expected. Furthermore, the performance of VG_Giraffe evaluated here is also much worse than that reported in tomato, another plant species (Fig. 1c,d of https://www.nature.com/articles/s41586-022-04808-9). This could further indicate that the variant dataset used in the evaluation might not be representative and accurate.

Line 333-335, "The EVG pipeline starts with the selection of the most suitable software according to the reference genome size, sequencing data quality, type and number of variants, and software preference": This is the core of the EVG pipeline, while the rules for selecting the software was not clearly described. The description in the method is also vague (Line 609-615). A table listing which genotypers will be selected for run in EVG under different reference genome sizes, sequencing data qualities/depths, type and number of variants... BTW, what is the exact meaning of "software preference" here?

For maize real dataset, only chr10 was analyzed according to Table S5. However, this was not mentioned in the manuscript, and it seems the entire genome was analyzed based on the CPU time (Fig. S19c). Please make this clear.

Table 1 and Table S1 are largely identical except that Table S1 contains information for four additional genotypers. Table 1 can be removed.

It would be more readable if some figures are converted to tables. Alternatively, a supplementary table lists the precision, recall and F-measure values for all the tests would be very helpful.

Line 385-396: This statement is wrong.

It is surprising that only 5.8 million SNPs were included in the maize genome graphs based on the simulated data (Table S4) as genomes of different maize cultivars are quite divergent. From the real data of 7 maize lines, 1.94 million SNPs were identified on the smallest chromosome (chr10, ~7% of the maize genome; Table S5), equivalent to ~28 million in the entire maize genome. These numbers are highly inconsistent. In addition, the numbers of SVs in all real datasets (Table S5) are unexpectedly low.

**Authors’ response**

The main changes include: 1. To explain the observations that the genotyping performance of some tools in plant ge nomes got worse when more genomes graphed and was not good like in human genomes,  we calculated the repetitiveness for plant and human genomes, as well as their genome  graphs. As a response, we also did a similar evaluation of these tools using the human  genome dataset. Additionally, we added some descriptions to clarify the performance ro bustness and advantages of some graph-based tools in certain types of variants. 2. As a comparison baseline, we added tests for three widely used variant genotyping tools  (GATK, DeepVariant, and Delly) based on linear reference genomes.

3. We now run EVG for each simulated data set, as we did for other graph-based genotyping  tools.

4. For the simulated maize data, we simulated much more variants. To finish the evaluation  in a limited time, we evaluated the genotyping performance only for chromosome 10. 5. We also conducted a leave-one-out experiment to evaluate the performance of genome  graph tools.

6. As *Brassica napus* is allopolyploid, we replaced it with *Glycine max* for variant genotyp ing evaluation in diploid plant genomes with a more comprehensive range of genome size.

Below, we provide a point-by-point response to the reviewers' comments, with responses indi cated in blue.

**Reviewer 1**

Focusing on the genotyping issue in plants, the authors conducted a comprehensive benchmark ing study to evaluate the performance of eight selected genotyping software, taking into account  various factors that can influence genotyping outcomes in plants. Based on the benchmarking  results, the authors identified the most suitable software for different scenarios, which were  then integrated into a package with an internal pipeline. This package enables researchers to  genotype all types of variants in any plant species, with reduced requirements for computational  resources and sequencing data. The authors effectively addressed the significant and practical  topic of modern genotyping in plants by evaluating and integrating existing tools. Their thorough understanding of the issue is evident through their in-depth investigation of relevant tools,  algorithms, and other aspects. The benchmarking work is well-designed, supported by authentic  data, and the developed package meets expectations as a product. The manuscript is enriched  with informative figures and tables. In summary, this manuscript represents valuable and excellent work. However, there are still some issues in the manuscript that need to be addressed  and improved before its publication.

1. The main result claimed in title is "a comprehensive benchmark of … algorithms", but the  benchmarking is actually done on several tools with overlapped algorithms, and less algorithm  anatomy/analyze is given through the manuscript. I suggest the author to enhance the descrip tion of the innovative algorithm in the EVG.

Response 1: Thank you very much for your suggestion. In this study, we already have collected  all free-available graph-based genotyping tool so far. We agree that they share some common  algorithms. However, they also have some differences in indexing, mapping strategies (kmer based or read-based), mapping algorithms and genotyping models. The algorithm comparison  of these tools has been described in the Introduction section **(lines 59-77)**, summarized in **Ta bles S1**, and discussed in **lines 443-470**. In the revision, we now add algorithm descriptions in  the manuscript **(lines 422-442)** to explain why genome graph software performs poorly in  plants. Additionally, as you suggested, we have provided a more detailed description of EVG  in the Methods section **(lines 655-678)**.

2. I didn't see how variant with differed coordinates from each genotypers are merged. This is  critical to the pipeline.

Response 2: Thank you for bringing that to our attention. To address the issue caused by incon sistent coordinates of the same variants from different software, EVG employs a clustering  approach based on variant size and position to cluster the output (EVG merge). A schematic  figure **(Fig. S20)** shows the processing.

For variants larger than 50 bp, EVG clusters them together if their positional differences are  less than 200 bp. Similarly, for indels smaller than 50 bp, their positional differences should be  less than 10 bp, while SNPs require precise positional matching. In addition to considering  positional information, EVG also requires that the length difference ratio of variants within the  same cluster is less than 0.25.

Finally, EVG selects the most likely genotyping according to consistency across different  genotypers and depth information. For small variants, including SNPs and indels, the genotype  is determined as the one supported by most genotypers. When no genotype is supported by  more than one genotyper, EVG will skip this variant. For SV genotyping, EVG also chose the  genotype supported by most genotypers. In cases where all genotype frequencies are equal to  1, the genotype is one with the smallest normalized absolute depth **(lines 673-694)**.

3. The genome size of Brassica napus is approximately 1Gb. However, it is an allopolyploid.  Its heterozygosity differs significantly from several other species. In order to achieve a more  comprehensive range of genome size, I suggest incorporating another diploid species with a  genome size of 1G.

Response 3: Thanks. We fully agree that it is greatly challenging to genotype allopolyploid  *Brassica napus* due to the “heterozygosity” or sequence similarity between homologous sub genomes. In the revised manuscript, we have replaced *Brassica napus* with the diploid genome *Glycine max*, which allows us to maintain consistency with other species. In the revised manu

script, we have included the genotyping results of *Brassica napus* as supplementary information,  which can be found in **Figure S2**.

4. (line 66) The vg toolkit developed by Erik Garrison's team is officially denoted in all lower case (together with its subprograms). Better to mention it as "vg map" "vg giraffe" without  hyphen. (All upper case "VG" exists some places in the docs, exhibiting little inconsistency.  But largely and especially in publication it's presented as "vg".)

Response 4: Thanks. In the revised manuscript, we have corrected them into "vg map" and "vg  giraffe".

5. (line 180) Read depth is an indicator other than genotype quality, thus the relation should be  "substitution when lack" rather than "proxy". Moreover, DP and GQ are not presented in the  figure, so abbreviation should belong to elsewhere in the writing (line 134 as their first pres ence).

Response 5: Thank you for pointing this out. We have now changed "proxy" to "substitution"  and added the abbreviations for genotype quality and read depth to the main text **(lines 129- 130)**.

6. (line 264) "breakpoint deviations" is mentioned several times in this section, but a concise  definition is lacking.

Response 6: Breakpoint deviations refer to the distance between the real SV breakpoints and  the observed SV breakpoints resulting from SV callers. This can lead to mapping errors and impact the following genotyping for those variant genotyping tools. In the revised manuscript,  we have added the definition for “breakpoint deviations” **(lines 273-275)**.

7. (line 284) The variant length is clarified with a formula in parenthesis, however it may be come misleading. In fact, I took some while to confirm that it is a minus expression rather than  two phrase connected with hyphen.

Response 7: Thank you. We have now replaced it with "alternative allele length minus reference  allele length" **(line 293)**.

8. (line 340-341) "when sequencing data is high enough". How much coverage is enough? Response 8: Sorry for this confusion. EVG defaults to a sequencing depth of 15×, and if the  depth is greater than 15×, EVG will downsample 15×reads for subsequent genotyping. In the  revised manuscript, we have clarified this **(line 354)**.

9. The legend of Figure 5b may be confusing, especially the term "presence". Presence variation? Response 9: In this manuscript, for “presence” and “genotype”, we follow the same definition  as used in the vg giraffe paper [1]."presence" refers to the detection of SVs regardless of their  genotype, while "genotype" requires both the detection of the SVs and agreement between its  genotype and the true genotype. In the revised manuscript, we now provide an explanation  **(lines 330-333)**.

10. To achieve consistent result with the author have tested through, the version and parameters  of the involved tools should be locked and adhere to versioning of the main program EVG  (source code).

Response 10: In the revised manuscript, we have explicitly stated the software version **(lines  530-535)**. Additionally, we have provided comprehensive documentation of the detailed pa rameters on our GitHub page: https://github.com/JiaoLab2021/EVG/wiki/EVG-paper.

**References**

1. Siren J, Monlong J, Chang X, Novak AM, Eizenga JM, Markello C, Sibbesen JA, Hickey  G, Chang PC, Carroll A, et al: **Pangenomics enables genotyping of known structural  variants in 5202 diverse genomes.** *Science* 2021, **374:**abg8871.

**Reviewer 2**

The paper consists of two parts (1) a comprehensive evaluation of eight graph-based geno types, and the introduction on an "ensemble" genotyper EVG that uses information from all  either genotypers to decide the best answer for each variant. This is shown to have overall better  F-measure than all eight ones individually.

Overall, I find the paper interesting: via its first part it helps biologists understand the per formance of state-of-the-art genotypes, and via its second part it proposes a method to get the  best answer from all, for overall better accuracy, and which works even with 5x coverage.

However, I have some questions and comments that would need to be answered before con firming the above conclusions. My major comment is that I did not find a strong motivation on  why we should be using a graph-based genotyper in the first place for complex plant genomes.  For example, if I understood correctly, the ground-truth for benchmarking the real dataset was  constructed using single reference(s), which to me it implies that the paper trusts more geno typing using single reference(s) than graph-based ones.

Response: Thank you very much for reviewing our manuscript. Currently, many plant popula tion sequencing data are generated using second-generation sequencing technologies. The  downstream analysis, such as genome-wide association studies (GWAS), relies on the accurate  genotyping of SNPs and indels from these short reads. However, it is important to note that  SVs can have a great impact on trait-associated genes or non-coding sequences. Unfortunately,  it is very challenging to accurately detect and genotype SVs only using a linear reference ge nome and short-read data, especially in complex plant genomes. In practical pan-genome stud ies with hundreds or thousands of individuals, only several representative individuals will be  sequenced using long reads, which can provide a high-quality structural variation dataset, and  others will be only sequenced with short reads. We think that this is one of the most suitable  scenarios in which graph tools can be utilized.

However, for graph-based genotyping tools, there was no comprehensive evaluation in  plants as we did in this study, although they presented advantages in previous studies on human  genomes. We believe our benchmarking study can help user decide which graph-based geno typing tool might be more helpful for their genotyping studies.

By the way, for the ground-truth real dataset, we clarify this in detail in the below response.

Specific comments:

1. As far as I understand, the conclusion of section "Effects of genome number in the graph" is  that the more genomes in the graph, the worse the results. While I agree that the larger this  number, the more complex the graphs become, and the harder the alignment problem can be come, this appears to me as counter-intuitive to the whole reason of building a pan-genome  graphs and calling variants there. If alignment gets worse, then why work with genome graphs  for plant genomes in the first place?

Response 1: Many thanks for your comment. We agree that it sounds counter-intuitive. But we  first would like to point out that some tools still presented relatively stable performance **(Re sponse Table 1**, as the red numbers indicate**)** as more genomes were incorporated into the ge nome graph. We do apologize that we might convey the misleading impression that all graph  tools performed worse in all types of variants when more genomes were graphed. We now  clarify this at **lines 254-255**.

We would also like to clarify that the first point of this study is to figure out the perfor mance of currently available graph-based genotypers in plant genomes. As our evaluation re vealed that there were still many challenges, including the issue of “worse performance at more  genomes” and performance differences for current graph-based genotyping tools, we were mo tivated to develop our genotyping pipeline, EVG. And this EVG pipeline reached much more  stable performance **(Fig. 3 and Response Table 1)** when more genomes were graphed. Addi tionally, EVG showed much higher recall and precision for SV genotyping compared to the  single reference-based genotyping tools **(Fig. S4)**. So, we suggested users try our EVG pipeline  when doing variant genotyping.

Besides, we also conducted the evaluation based on the previously published real human  variant dataset [2]. We also found that the performance of some tools for certain types of vari ants got worse when more genomes were graphed, while other tools presented stable or even  higher F-scores **(Response Table 2)**.

As a comparison, we also include three widely used single reference based genotyping  tools, including GATK [3], DeepVariant [4], and Delly [5], to do variant genotyping. This anal ysis result is now described in manuscript **lines 178-186** and **Figure S4**. Compared to those  graph-based genotyping tools, we found these linear reference-based tools had very close per formance for small variants but got worse results for structural variants. Therefore, we believe  genome graphs based genotyping methods have significant advantages for large variants.

Response Table 1. F-scores under different number of graphed A. thaliana genomes based

on simulated data.

**Figure S4. The performance of variant callers based on linear reference genome and genome  graph for *A. thaliana*, *Oryza sativa*, *Glycine max*, and *Zea mays*.** Variants from seven individuals  of *A. thaliana*, *Oryza sativa*, *Glycine max*, and *Zea mays* genomes were used for genome graph  construction. 30× paired-end (2×150 bp) short reads were simulated for variant genotyping. For  each genotyping scenario, the highest F-measure value is labeled. Transparent and solid bars repre

sent the ability to predict variant “presence” (detection of variant regardless of the genotype) and  exact “genotype” (requires both the detection of the variant and agreement between its called  genotype and the true genotype).

2. Can we conclude that genome graphs are a helpful tool for genomes such as human, and not  for plant genomes? If so, I find this to be a major drawback for this work, because then one  should just to variant calling using single reference(s).

Response 2: We agree that the single reference genome has similar or very close performance  for genotyping small variants especially SNPs in plants according to our expanded evaluation  including the experiments for single reference based genotyping tools. However, for the large  variants especially the structural variants, our evaluation revealed that graph-based genotyping  tools and our EVG pipeline had much better performance compared to single reference based  methods **(lines 178-186, Fig. S4)**.

We are afraid that we cannot agree with you that this is a drawback, although the perfor mance in plants is not as good as in human genomes. Plant genomic researchers might choose

graph tools for variant genotyping because these tools have been proven to have advantages for human genomes. However, no comprehensive evaluation of these tools has been done in plants  as we did in this study. We believe our benchmarking study can help user decide which graph

based genotyping tool might be more helpful for their genotyping studies.  Besides, regarding the different performance between human and plant genomes, we have  done comparisons in terms of genome repetitiveness analyses with the single reference genome  and genome graphs for both human and plant genomes. We found that plant genome graphs  were enriched with repetitive k-mers, which prevented the short reads from being mapped cor rectly **(lines 422-442 and Fig. S25)**. This can be the explanation for the performance differ ences between humans and plants. Also, this should be considered for the development of  graph-based tools in the future. Here we describe the genome repetitiveness analysis in detail below.

We first calculated the repetitiveness by comparing non-unique k-mers to the total number  of k-mers in the whole genome assembly sequences **(Fig. S25a)**, as the degree of k-mer unique ness will affect the genotyping performance of graph-based tools. For all genomes tested in this  study except *A. thaliana*, they have relatively higher repetitiveness compared to the human  genome. In particular, non-unique k-mers in the maize genome account for up to 68%, while  the larger human genome only contains 18% non-unique k-mers. Moreover, we calculated the  frequency of k-mers in each node of the genome graph constructed from real data **(Fig. S25b)**.  The proportion of specific k-mers (k-mers with a frequency of 1 in the node) was highest in the  human genome graph (62%), which means the short reads from the human genome are rela

tively easier to be mapped correctly.

This genome complexity challenges the genotyping of plant genome graphs. Graph-based  genome software such as PanGenie [2], often loads only specific k-mers into memory for gen otyping to improve accuracy and control memory consumption. For example, in the maize ge nome graph, only 17% of k-mers had a node frequency of 1, which means that only 17% of k mer information could be used for genotyping. Therefore, when there are not enough specific  k-mers for a variant, the genotyping performance will be poor.

**Figure S25. The k-mer based comparisons of genome repetitiveness between plants and hu man based on single reference genomes (a) and (b) the node frequency of k-mers in the genome  graph.** Repetitiveness was assessed by calculating the ratio of non-specific k-mers to the total num ber of k-mers in the reference genome. The frequency of k-mers in nodes refers to the number of  occurrences of k-mers in all nodes of the genome graph. The variation data for *A. thaliana*, rice, and  maize were obtained from real population variation data used in this study. The human variation

data were obtained from the variants identified in 11 diploid populations in the PanGenie paper [2].  The number in parentheses represents the number of genomes used.

3. Following up on the above point, I think you should also include in your evaluation (both in  part (1) and part (2)) a couple of state-of-the-art variant callers working with only reference  genomes (not graphs). This would be the baseline, and this would motivate using genome  graphs in the first place for such complex genomes.

Response 3: Thank you very much for the suggestion. We have added some analyses of geno typing only using a single linear reference and short reads. For SNP and indels (<50 bp) geno typing, we utilized the state-of-the-art tools GATK [3] and DeepVariant [4]. Both of them have  been widely used in variant calling for human and plant genomes. For SV genotyping, we also  used a very popular tool, Delly [5]. We have run them to evaluate their performance on simu lated data for all tested plant genomes. New results have been incorporated into **Figure S4**. The  detailed description can be found in **lines 178-186**.

Briefly, the single reference genome has similar or very close performance for genotyping  small variants especially SNPs in plants. However, for the large variants especially the struc tural variants, our evaluation revealed that graph-based genotyping tools and our EVG pipeline  had much better performance compared to single reference based methods.

4. I did not find how from some graph aligners (e.g. Graph Aligner) you call variants. I quickly  checked the GraphAligner manual (https://github.com/maickrau/GraphAligner) and I did not  find some option that it would support this. If you use an external tool, could it introduce some  bias?

Response 4: Yes, GraphAligner is just a genome graph aligner. We use the alignment results  from GraphAligner and do variant genotyping with vg. GraphAligner employs different align ment algorithms as compared to vg map and vg giraffe. As the common graph-based genotyp ing pipeline include two steps, one for read mapping and the other for genotyping, we include  this GraphAligner based genotyping pipeline to see whether the mapping step affects the final  performance of genotyping.

5. I do not understand how the plots in Figs 1, 2, ... are made. You say "For each genotyper,  precision is plotted against recall as the genotyping quality threshold varies." Can you explain  more precisely?

Response 5: When performing genotyping, the software calculates a quality score for each gen otyping result. We filter the results continuously based on the descending order of the quality  scores. At each filtering step, we calculate the recall rate and accuracy under the corresponding  threshold. Through this process, we can generate a series of recall rates and accuracy values  and plot their precision-recall curves on the same graph for comparison.

6. I also have a major comment about the way the experimental results in both parts of the paper  are presented. In part (1) you express these as ROC curve where precision and recall are directly  visible. In part (2) you combine these into F-measure, but potential users cannot tell e.g. how  precise EVG is. I think there should be a uniform evaluation.

Response 6: Yes, using F-score to evaluate may indeed prevent people from accurately obtain ing recall and precision information. In the revised version of the manuscript, we have added  the results of EVG to the ROC curve **(Fig. 1)**.

7. Following up on the above, in part (1) you have extensive experimental scenarios, but in part  (2) you are not very extensive in experimenting with your tool EVG. I find this to be a major  issue: if you have a comprehensive evaluation of eight genotypers, why not test your novel  genotyper in the same comprehensive evaluation? A way to address this could be to describe  EVG before the evaluation, and include EVG in all scenarios of the comprehensive evaluation. Response 7: Thank you very much for your suggestion. As you suggested, we have run our tool  EVG to do all types of variants genotyping for all experimental scenarios. More evaluation for  EVG include the performance of variant genotyping for rice, soybean, maize, and *Brassica  napus* using 30× sequencing data with 2×150 bp read length. Briefly, EVG performs better than  other software in rice **(Fig. 1d-1f)**, soybean **(Fig. 1g-1i)**, maize **(Fig. 1j-1l)**, and *Brassica napus* **(Fig. S2)**, especially in terms of maintaining good performance for all types of variations.

We also agree that it can be a good alternative way to describe EVG before the evaluation.  However, the motivation for developing our EVG pipeline is based on the evaluation. We real ize that we develop a new pipeline just because these current graph-based genotypers are not  so efficient in plant genomes as in human genomes.

8. In part (2), could you give more technical details on the methods of EVG? For example,  section "Step 3: merge and finalize genotyping" could be enhanced with one or two figures and  some more explanations.

Response 8: Thank you very much for your good suggestion. To address the issue caused by  inconsistent coordinates of the same variants from different software, EVG utilizes a clustering  approach based on variant size and position to merge the output (referred to as EVG merge) **(Fig. S20)**. For variants larger than 50 bp, EVG clusters them together if their positional differ ences are within 200 bp. Similarly, for Indels smaller than 50 bp, their positional differences  should be less than 10 bp, while SNPs require precise positional matching. Additionally, EVG  considers the length difference ratio of variants within the same cluster, requiring it to be less  than 0.25.

Finally, EVG selects the most likely genotyping according to consistency across different  genotypers and depth information. For small variants, including SNPs and indels, the genotype  is determined as the one supported by most genotypers. When no genotype is supported by  more than one genotyper, EVG will skip this variant. For SV genotyping, EVG also chose the  genotype supported by most genotypers. In cases where all genotype frequencies are equal to  1, the genotype is the one with the smallest normalized absolute depth. **(lines 673-694)**.

**Figure S20. The process of merging and finalizing genotype in the EVG pipeline.**

9. Could you give a more intuitive explanation of how come your tool is overall better than all  other genotypes, by not using any novel insights? Is it that it takes the "best" answer for each  variant, and thus it is overall better?

Response 9: As shown in **Figure S17**, there is a strong complementarity among different ge nome graph software, meaning that although one software may have incorrect genotyping at  certain loci, other software can accurately genotype those loci. It is this observation that led us  to develop EVG to leverage this characteristic. As described in **Response 8**, EVG selects the  most probable genotype supported by the most tools, ensuring the accuracy of genotyping. If  more than one candidate genotypes exist, EVG determines the most likely result based on the  depth information of the genotype. In other words, the closer the genotype's depth is to the  average depth, the higher its likelihood. Too high or too low depths may indicate false positives.

**Figure S17. The intersection of correct genotyping results among different software.** The num ber of intersections for a specific combination is shown above the column. The number and propor tions are provided in parentheses at the bottom. **(a)** The intersection among different software for  genotyping SNPs and indels. **(b)** The intersection among different software for genotyping SVs.

10. In Sec "Performance on real data", you construct a ground-truth for this real dataset by  aligning the reads to a single reference (if I understand correctly). But then again this defeats  the message of the paper: if you consider single-reference alignments as ground-truth, then why  should we do graph-based genotyping in the first place?

Response 10: We would like to clarify the method we used for constructing the ground-truth  variant dataset. Briefly, for SNPs and indels (<50 bp), we used the filtered results called by  both the GATK and BCFtools based on the single reference and short reads (>100 bp). With  this intersecting and filtering, a high-quality variant data set can be obtained. We did not use  long reads for identifying small variations due to indel enriched errors in third-generation long  reads from these tested plant genomes. For structural variants, we integrated the third-genera

tion long reads-based mapping and long read based genome assembly comparisons. The long  reads and genome assembly have great advantage for variant calling especially the structural

variations as reported in previous studies [6-9]. And the long reads-based ground-truth con structing approach has also been employed in some benchmarking studies or tool performance  evaluation [10, 11]. For example, the pan-genome study of tomato, the authors identified vari ations smaller than 50 bp by the linear reference tool DeepVariant and detect SVs based on  long reads mapping [12].

We evaluate all graph-based genotypers only based on short reads, as we often have no  long reads for population resequencing study, and we often want to do genotyping in a cost effective way only using short reads. In practical pan-genome studies with hundreds or thou sands of individuals, only several representative individuals will be sequenced using long reads  which can provide a high-quality of structural variation dataset, and other will be only se quenced with short reads. And this is the most suitable scenario that graph tools can be utilized.

11. Even more critical, if in part (1) you show that graph-based genotypers are more accurate  than ones based on single reference(s), then why should we construct the ground-truth for real  data using genoetypers based on single reference(s)?

Response 11: Like **Response 10,** we employed different strategies to construct the ground truth  set for different types of variants in our real data analysis. The integration of third-generation  sequencing data and the genome assembly comparison can be highly effective in identifying  SVs.

However, one challenge in many population-level genomic or pan-genome studies is that  huge number of individuals are re-sequenced using second-generation sequencing technologies.  Therefore, when trying to obtain SV genotypes from each individual, it becomes difficult to  use a linear reference genome **(Fig. S4)**. The genome graph tools can potentially address this  challenge as our evaluation showed in this study, compared to those linear reference based  genotyping tools. However, it should be noted that there are still many challenges in applying  genome graphs to plants, and further research efforts will be needed in this direction in the  future.

**Figure S4. The performance of variant callers based on linear reference genome and genome  graph for *A. thaliana*, *Oryza sativa*, *Glycine max*, and *Zea mays*.** Variants from seven individuals  of *A. thaliana*, *Oryza sativa*, *Glycine max*, and *Zea mays* genomes were used for genome graph  construction. 30× paired-end (2×150 bp) short reads were simulated for variant genotyping. For  each genotyping scenario, the highest F-measure value is labeled. Transparent and solid bars repre

sent the ability to predict variant “presence” (detection of variant regardless of the genotype) and  exact “genotype” (requires both the detection of the variant and agreement between its called  genotype and the true genotype).

**References**

1. Siren J, Monlong J, Chang X, Novak AM, Eizenga JM, Markello C, Sibbesen JA, Hickey  G, Chang PC, Carroll A, et al: **Pangenomics enables genotyping of known structural  variants in 5202 diverse genomes.** *Science* 2021, **374:**abg8871.

2. Ebler J, Ebert P, Clarke WE, Rausch T, Audano PA, Houwaart T, Mao Y, Korbel JO, Eichler  EE, Zody MC, et al: **Pangenome-based genome inference allows efficient and accurate  genotyping across a wide spectrum of variant classes.** *Nat Genet* 2022, **54:**518-525.

3. McKenna A, Hanna M, Banks E, Sivachenko A, Cibulskis K, Kernytsky A, Garimella K,  Altshuler D, Gabriel S, Daly M, DePristo MA: **The Genome Analysis Toolkit: a  MapReduce framework for analyzing next-generation DNA sequencing data.** *Genome  Res* 2010, **20:**1297-1303.

4. Poplin R, Chang P-C, Alexander D, Schwartz S, Colthurst T, Ku A, Newburger D, Dijamco  J, Nguyen N, Afshar PT, et al: **A universal SNP and small-indel variant caller using deep  neural networks.** *Nat Biotechnol* 2018, **36:**983-987.

5. Rausch T, Zichner T, Schlattl A, Stutz AM, Benes V, Korbel JO: **DELLY: structural  variant discovery by integrated paired-end and split-read analysis.** *Bioinformatics* 2012,  **28:**i333-i339.

6. Gao Y, Yang X, Chen H, Tan X, Yang Z, Deng L, Wang B, Kong S, Li S, Cui Y, et al: **A  pangenome reference of 36 Chinese populations.** *Nature* 2023, **619:**112-121. 7. Liao WW, Asri M, Ebler J, Doerr D, Haukness M, Hickey G, Lu S, Lucas JK, Monlong J,  Abel HJ, et al: **A draft human pangenome reference.** *Nature* 2023, **617:**312-324. 8. Zook JM, McDaniel J, Olson ND, Wagner J, Parikh H, Heaton H, Irvine SA, Trigg L, Truty  R, McLean CY, et al: **An open resource for accurately benchmarking small variant and  reference calls.** *Nat Biotechnol* 2019, **37:**561-566.

9. Zook JM, Hansen NF, Olson ND, Chapman L, Mullikin JC, Xiao C, Sherry S, Koren S,  Phillippy AM, Boutros PC, et al: **A robust benchmark for detection of germline large  deletions and insertions.** *Nat Biotechnol* 2020, **38:**1347-1355.

10.Shang L, Li X, He H, Yuan Q, Song Y, Wei Z, Lin H, Hu M, Zhao F, Zhang C, et al: **A  super pan-genomic landscape of rice.** *Cell Res* 2022, **32:**878-896.

11.Zhang F, Xue H, Dong X, Li M, Zheng X, Li Z, Xu J, Wang W, Wei C: **Long-read  sequencing of 111 rice genomes reveals significantly larger pan-genomes.** *Genome Res* 2022, **32:**853-863.

12.Zhou Y, Zhang Z, Bao Z, Li H, Lyu Y, Zan Y, Wu Y, Cheng L, Fang Y, Wu K, et al: **Graph  pangenome captures missing heritability and empowers tomato breeding.** *Nature* 2022,  **606:**527-534.

13.Jiao WB, Schneeberger K: **Chromosome-level assemblies of multiple Arabidopsis  genomes reveal hotspots of rearrangements with altered evolutionary dynamics.** *Nat  Commun* 2020, **11:**989.

14.Hufford MB, Seetharam AS, Woodhouse MR, Chougule KM, Ou SJ, Liu JN, Ricci WA,  Guo TT, Olson A, Qiu YJ, et al: **De novo assembly, annotation, and comparative analysis  of 26 diverse maize genomes.** *Science* 2021, **373:**655-662.

15.Liu Y, Du H, Li P, Shen Y, Peng H, Liu S, Zhou GA, Zhang H, Liu Z, Shi M, et al: **Pan Genome of Wild and Cultivated Soybeans.** *Cell* 2020, **182:**162-176 e113. 16.Song JM, Guan Z, Hu J, Guo C, Yang Z, Wang S, Liu D, Wang B, Lu S, Zhou R, et al:  **Eight high-quality genomes reveal pan-genome architecture and ecotype  differentiation of Brassica napus.** *Nat Plants* 2020, **6:**34-45.

17.Kosugi S, Momozawa Y, Liu X, Terao C, Kubo M, Kamatani Y: **Comprehensive  evaluation of structural variation detection algorithms for whole genome sequencing.** *Genome Biol* 2019, **20:**117.

**Reviewer 3**

The authors provide a comprehensive benchmark and comparison of genome-graph-based gen otypers on plant genomes. Given that most of these methods were originally developed for the  human genome, their performance on plant genomes remains unclear. The benchmark and com parison presented in this manuscript offer useful insights.

Additionally, the authors propose an ensemble-based genotype calling approach by combining  different genotypers and demonstrate, through simulation data, that the ensemble approach per forms better. I have the following comments on the experiment design and results, for which I  hope the authors can provide further data support.

Major:

1. One major assumption made in this study is that graph-genome-based genotyping outper forms linear reference-based approaches. However, it appears that the performance in SNP and  INDEL genotype calling is less favorable compared to earlier results reported by GATK and  DeepVariant. I wonder whether this discrepancy is due to the nature of the data being sourced  from plant genomes or if it indicates a general limitation of the graph-genome approach in  SNP/INDEL genotyping. To address this, it would be beneficial if the authors could provide  additional supporting results and analyses.

Response 1: Many thanks for your comment. In the revised manuscript, we have evaluated the  performance of linear reference based genotyping tools. For SNP and indels genotyping, we used GATK [3] and DeepVariant [4]. **(Fig. S4)**. For SNP genotyping, GATK has F-score values  of 0.96 (*A. thaliana*), 0.99 (rice), 0.93 (soybean) and 0.77 (maize), while DeepVariant has val ues of 0.98, 0.99, 0.96, 0.87. For indel genotyping, GATK has F-score values of 0.86 (*A. thali ana*), 0.81 (rice), 0.90 (soybean) and 0.45 (maize), while DeepVariant has values of 0.90, 0.87,  0.92, 0.63. The graph-based tools BayesTyper, Paragraph, GraphTyper2, and EVG presented a  bit higher or close F-scores, while tools such as vg map, vg giraffe, and PanGenie showed a  relatively lower F-scores, compared to GATK and DeepVariant.

We agree that the performance in SNP and indels genotyping in plant is less favorable for  some graph tools compared to studies in human genomes. To provide additional supporting  results about this discrepancy, we have compared the genome repetitiveness between human  genome and plant genomes.

We calculated the repetitiveness by comparing non-unique k-mers to the total number of k mers in the whole genome assembly sequences **(Fig. S25a)**, as the degree of k-mer uniqueness  will affect the genotyping performance of graph-based tools. For all genomes tested in this  study except the *A. thaliana*, they have relatively higher repetitiveness compared to the human  genome. In particular, non-unique k-mers in the maize genome account for up to 68%, while  the larger human genome only contains 18% non-unique k-mer.

Moreover, we calculated the frequency of k-mers in each node of the genome graph con structed from real data for *A. thaliana*, rice, and maize and human genomes **(Fig. S25b)**. We  observed that the proportion of specific k-mers (k-mers with a frequency of 1 in the node) was  highest in the human genome graph (62%). However, the proportions of specific k-mers in  plant genome graphs was much lower (maize: 17%, rice: 36%, *A. thaliana* 24%). These k-mer

comparisons suggests that smaller plant genomes can often have higher complexity compared  to human genomes.

This genome complexity challenges the genotyping in plant genome graphs. Graph-based  genome software such as PanGenie [2], often loads only specific k-mers into memory for gen otyping to improve accuracy and control memory consumption. For example, maize genome only 17% of k-mers had a node frequency of 1, which means that only 17% of k-mer infor mation could be used for genotyping. Therefore, when there are not enough specific k-mers for  a variant, the genotyping performance will be poor.

Furthermore, software based on alignment indexing methods relies on variations of BWT,  suffix arrays, or k-mer indexing algorithms, such as GBWT, GCSA2, or minimizer. When there  are too many repetitive sequences in the genome, it significantly affects sequence alignment in  terms of accuracy and resource consumption. This issue becomes more severe as more genomes are graphed. In the revised manuscript, we have added an explanation of this phenomenon **(lines  422-442)**.

**Figure S4. The performance of variant callers based on linear reference genome and genome  graph for *A. thaliana*, *Oryza sativa*, *Glycine max*, and *Zea mays*.** Variants from seven individuals  of *A. thaliana*, *Oryza sativa*, *Glycine max*, and *Zea mays* genomes were used for genome graph  construction. 30× paired-end (2×150 bp) short reads were simulated for variant genotyping. For

each genotyping scenario, the highest F-measure value is labeled. Transparent and solid bars repre sent the ability to predict variant “presence” (detection of variant regardless of the genotype) and  exact “genotype” (requires both the detection of the variant and agreement between its called  genotype and the true genotype).

**Figure S25. The k-mer based comparisons of genome repetitiveness between plants and hu man based on single reference genomes (a) and (b) the node frequency of k-mers in the genome  graph.** Repetitiveness was assessed by calculating the ratio of non-specific k-mers to the total num ber of k-mers in the reference genome. The frequency of k-mers in nodes refers to the number of  occurrences of k-mers in all nodes of the genome graph. The variation data for *A. thaliana*, rice, and  maize were obtained from real population variation data used in this study. The human variation  data were obtained from the variants identified in 11 diploid populations in the PanGenie paper [2]. The number in parentheses represents the number of genomes graphed.

2. The evaluation of the proposed ensemble approach, EVG, is exclusively benchmarked on  simulation data. I wonder how well the performance of the EVG approach when applied to real  data from the human genome. There are high-quality benchmark datasets available from the  Human Pan-Genome Project (https://www.nature.com/articles/s41586-023-05896-x#data

availability), which includes T2T haplotype-resolved assemblies and 30X whole-genome se quencing (WGS) short reads that are publicly available. Incorporating such real-world data into  the evaluation could provide valuable insights into the practical utility of the EVG method. Response 2: Thanks for your kindly suggestion. We total agree that EVG should be evaluated  on more real data. Actually, we have assessed EVG’s performance based on real data from *A.  thaliana*, rice, maize, and apricot **(Fig. 6 and Fig. S22)**. The Human Pangenome Reference  Consortium (HPRC) dataset is indeed a very valuable resource, but the high number of variants  it contains meant that we did not have sufficient computational resources to analyze it within  the given time. Instead, we evaluated the performance of EVG using real human genome data  from the PanGenie paper [2].

As shown in **Response Figure 1**, EVG performs well on the human genome, although it  has not outperformed PanGenie for indels and insertions. Nevertheless, compared to other soft ware, EVG still demonstrates advantages. Since these published graph-based genotyping tools  are primarily developed for the human genome, they generally perform well in human genomes.  In contrast, in plant genomes, where these published tools do not perform as well as in human  genomes, EVG can leverage the advantages of different software tools to achieve better geno typing performance.

**Response Figure 1. The genotyping performance of graph-based genotypers for the human  genome.** For each genotyper, the genome graph was constructed based on the genetic variations  from eleven human genomes as used in the PanGenie paper [2]. To evaluate each genotyper’s per formance, we used 30× paired-end Illumina short reads from a human sample NA24385 [2].

3. The selected variations for the simulation dataset have been curated from cohort-released  results primarily based on short reads. It is noteworthy that a substantial number of variations  are not identified when compared to results obtained from long reads. For instance, in the case  of polymorphic transposons within the reference genome, most long-read callers can accurately  identify them as deletions, whereas short-read callers often miss or misidentify them. Despite  the inherent challenges in identifying such variations from short reads, it would be beneficial  to assess the performance of graph-based genotypers in accurately genotyping these instances.  This additional evaluation would provide valuable insights into the effectiveness of graph based methods in handling challenging scenarios like polymorphic transposons in short-read  data.

Response 3: Thank you very much for your suggestion. We fully agree that long reads can be  very helpful for identifying variations especially those transposons-related structural variations.  Actually, we have utilized the long reads and genome assemblies for constructing both simu lated and real variants data set (details included in the Method section, **lines 538-613**). For  example, the simulated variation set of *A. thaliana* is derived from genome-to-genome align ments, including seven genomes we previously published [13].

Regarding the genotyping SVs related to transposons or repeats, we have evaluated the  effects of different types of repeat sequences (including simple repeats, DNA transposons and retrotransposons) around breakpoints of large (>50 bp) deletions and insertions in *A. thaliana* genomes, which was described in **lines 311-319** and summarized in **Figure 4d and 4e**. Other  evaluation of the repeats’ effects on genotyping was shown in **Figure S7**. These evaluations

revealed that the genotyping performance of all graph-based tools was reduced in the repetitive  regions compared to the non-repetitive regions. And the long terminal repeat (LTR) retrotrans posons had the greatest impact on genotyping.

Minor:

1. The colors in the Figures are close to each other. The authors should change to more distin guishable colors to enhance clarity and readability.

Response 1: Thanks. In the revised manuscript, we have updated the colors and added markings  on the lines to improve visibility and readability.

2. The mutations used for generating the simulation data, as well as the commands for running  each tool, should be provided to facilitate the reproducibility of the results. Response 2: Thank you for your suggestion. The detailed commands and parameters for run ning all tools used in our study have been uploaded to the GitHub website. They can be freely  accessed from the weblink https://github.com/JiaoLab2021/EVG/wiki/EVG-paper.

**References**

1. Siren J, Monlong J, Chang X, Novak AM, Eizenga JM, Markello C, Sibbesen JA, Hickey  G, Chang PC, Carroll A, et al: **Pangenomics enables genotyping of known structural  variants in 5202 diverse genomes.** *Science* 2021, **374:**abg8871.

2. Ebler J, Ebert P, Clarke WE, Rausch T, Audano PA, Houwaart T, Mao Y, Korbel JO, Eichler  EE, Zody MC, et al: **Pangenome-based genome inference allows efficient and accurate  genotyping across a wide spectrum of variant classes.** *Nat Genet* 2022, **54:**518-525.

3. McKenna A, Hanna M, Banks E, Sivachenko A, Cibulskis K, Kernytsky A, Garimella K,  Altshuler D, Gabriel S, Daly M, DePristo MA: **The Genome Analysis Toolkit: a  MapReduce framework for analyzing next-generation DNA sequencing data.** *Genome  Res* 2010, **20:**1297-1303.

4. Poplin R, Chang P-C, Alexander D, Schwartz S, Colthurst T, Ku A, Newburger D, Dijamco  J, Nguyen N, Afshar PT, et al: **A universal SNP and small-indel variant caller using deep  neural networks.** *Nat Biotechnol* 2018, **36:**983-987.

5. Rausch T, Zichner T, Schlattl A, Stutz AM, Benes V, Korbel JO: **DELLY: structural  variant discovery by integrated paired-end and split-read analysis.** *Bioinformatics* 2012,  **28:**i333-i339.

6. Gao Y, Yang X, Chen H, Tan X, Yang Z, Deng L, Wang B, Kong S, Li S, Cui Y, et al: **A  pangenome reference of 36 Chinese populations.** *Nature* 2023, **619:**112-121. 7. Liao WW, Asri M, Ebler J, Doerr D, Haukness M, Hickey G, Lu S, Lucas JK, Monlong J,  Abel HJ, et al: **A draft human pangenome reference.** *Nature* 2023, **617:**312-324. 8. Zook JM, McDaniel J, Olson ND, Wagner J, Parikh H, Heaton H, Irvine SA, Trigg L, Truty  R, McLean CY, et al: **An open resource for accurately benchmarking small variant and  reference calls.** *Nat Biotechnol* 2019, **37:**561-566.

9. Zook JM, Hansen NF, Olson ND, Chapman L, Mullikin JC, Xiao C, Sherry S, Koren S,  Phillippy AM, Boutros PC, et al: **A robust benchmark for detection of germline large  deletions and insertions.** *Nat Biotechnol* 2020, **38:**1347-1355.

10.Shang L, Li X, He H, Yuan Q, Song Y, Wei Z, Lin H, Hu M, Zhao F, Zhang C, et al: **A  super pan-genomic landscape of rice.** *Cell Res* 2022, **32:**878-896.

11.Zhang F, Xue H, Dong X, Li M, Zheng X, Li Z, Xu J, Wang W, Wei C: **Long-read  sequencing of 111 rice genomes reveals significantly larger pan-genomes.** *Genome Res* 2022, **32:**853-863.

12.Zhou Y, Zhang Z, Bao Z, Li H, Lyu Y, Zan Y, Wu Y, Cheng L, Fang Y, Wu K, et al: **Graph  pangenome captures missing heritability and empowers tomato breeding.** *Nature* 2022,  **606:**527-534.

13.Jiao WB, Schneeberger K: **Chromosome-level assemblies of multiple Arabidopsis  genomes reveal hotspots of rearrangements with altered evolutionary dynamics.** *Nat  Commun* 2020, **11:**989.

14.Hufford MB, Seetharam AS, Woodhouse MR, Chougule KM, Ou SJ, Liu JN, Ricci WA,  Guo TT, Olson A, Qiu YJ, et al: **De novo assembly, annotation, and comparative analysis  of 26 diverse maize genomes.** *Science* 2021, **373:**655-662.

15.Liu Y, Du H, Li P, Shen Y, Peng H, Liu S, Zhou GA, Zhang H, Liu Z, Shi M, et al: **Pan Genome of Wild and Cultivated Soybeans.** *Cell* 2020, **182:**162-176 e113. 16.Song JM, Guan Z, Hu J, Guo C, Yang Z, Wang S, Liu D, Wang B, Lu S, Zhou R, et al:  **Eight high-quality genomes reveal pan-genome architecture and ecotype  differentiation of Brassica napus.** *Nat Plants* 2020, **6:**34-45.

17.Kosugi S, Momozawa Y, Liu X, Terao C, Kubo M, Kamatani Y: **Comprehensive  evaluation of structural variation detection algorithms for whole genome sequencing.** *Genome Biol* 2019, **20:**117.

**Reviewer 4**

Du et al. describe the benchmark analysis of a set of graph-based tools in genotyping genetic  variants in plant genomes. Based on their analysis results, the authors developed an Ensemble  Variant Genotyper (EVG) that can integrate variants genotyped with different existing graph based genotypers, each of which displayed advantages in genotyping certain categories of var iants in plant genomes of different sizes and complexities.

1. When genotyping variants using a graph-based pan-genome, the samples are normally not  those that are used in the pan-genome graph construction. Therefore, the authors should conduct  the "leave-one-out" experiment to evaluate the performance of different genotypers, as de scribed in the PanGenie paper (https://www.nature.com/articles/s41588-022-01043-w). The  current approach used by the authors for evaluation could be biased.

Response 1: Many thanks for your suggestion. To our knowledge, all the graph-based genotyp ing tools evaluated in our study except PanGenie have not utilized haplotype information during  genotyping. PanGenie incorporates haplotype recombination rates during the genotyping pro cess, and this is why leave-one-out experiments were conducted in the PanGenie paper. Other wise, PanGenie would assign excessive weight to the two haplotype paths present in the sample.

Regarding your comment, we first conducted leave-one-out experiments for variant geno typing with the maize genome data from this manuscript. As shown in **Response Figure 2**,  when performing leave-one-out experiments, only the genotyping performance of PanGenie is  affected across the four types of variations (SNPs, indels, deletions, and insertions). The geno typing performance of other software remains relatively stable.

We also run these leave-one-out experiments with real human genome data from the Pan Genie paper [2]. Again, only PanGenie's genotyping performance was affected **(Response Fig ure 3)**. However, EVG remains stable in maize, while its performance decreases in the human  genome because it employed PanGenie for genotyping in human data.

**Response Figure 2. Leave-one-out experiments for variant genotyping with simulated maize  data.** The variation and short reads data was from this manuscript. 30× paired-end short reads were  simulated. For leave-one-out experiment, genetic variations from the maize sample “sim”, which  was to be genotyped, were excluded. "F-measure (F) All" refers to the result using all variants, while  "Adjusted F-measure (F) Leave-one-out" refers to the test results based on the genome graph with

out variants from the sample "sim".

**Response Figure 3. Leave-one-out experiments for variant genotyping with real human data.** The human variation data was identified from eleven individuals in the PanGenie paper [2]. 30×  short reads data from the individual NA24385 was used for variant genotyping. For leave-one-out  experiment, genetic variations from the sample NA24385, which was to be genotyped, were ex

cluded. "F-measure (F) All" refers to the result using all variants, while "Adjusted F-measure (F)  Leave-one-out" refers to the test results based on the genome graph without variantsfrom the sample  NA24385.

2. In the abstract (Line 37), the authors claimed that EVG has a better genotyping performance  while "without increasing resource consumption". This seems not true. Based on what the au thors stated in the manuscript, in most cases at least two genotypers will be run in EVG (Line  607-615). In the software homepage (https://github.com/JiaoLab2021/EVG), it says that "In  fast mode, only certain software is utilized to genotype SNPs and indels, while precise mode  employs all software to genotype all variants". This will definitely and substantially increase  computational resource consumption (especially for the precise mode). In fact, Fig. S19 clearly  shows that EVG consumed more CPUs than most of the other genotypers. Nonetheless, it is  not clear how EVG can achieve less computational resource consumption as it employs all  software to genotype all variants, e.g., Paragraph was at least included in EVG for the variant  genotyping (Line 609-611), then how could Paragraph consume more CPU than EVG in all  cases (Fig. S19)? Could this be due to the downsampling of the input data in EVG? If this is  the case, then the comparison is biased.

Response 2: Thank you very much for pointing out this issue. In the previous manuscript, the  statement that our software does not increase resource consumption was due to the default set ting of using only 15×data for downstream genotyping, which was sufficient to achieve good  results. We agree this might be not a fair comparison for other tools regarding the resource  consumption. In the revised manuscript, we have corrected the description about the resource  consumption of EVG **(such as lines 405-410)**. We also ran EVG with 30×data and updated the  resource consumption figure **(Fig. S23 and Fig. S24)** accordingly.

3. The author concluded that including more genomes in the graph would decrease the perfor mances of all genotypers (Line 231-263). This is counterintuitive as more genomes included in  the graph will improve the read mapping and therefore improve the variant genotyping (this is  why more and more studies are focusing on graph-based pan-genomes constructed from multi ple genomes). This could indicate that the variants and/or the simulated reads used for evalua tion might not be representative.

Response 3: We fully agree that the genome graph will theoretically improve the read mapping  and the variant genotyping. But we first would like to clarify that some tools still presented  relatively stable performance **(Response Table 1, as the red numbers indicate)** as more ge nomes were incorporated into the genome graph. We do apologize that we might convey the  misleading impression that all graph tools performed worse in all types of variants when more  genomes were graphed. We now clarify this at **lines 254-255**.

Besides, we also conducted the evaluation based on the previously published real human  variant dataset [2]. We also found that the performance of some tools for certain type of variants

got worse when more genomes were graphed, while other tools presented stable or even higher  F-scores **(Response Table 2)**.

Besides, our EVG pipeline utilized the different advantages of multiple graph-based geno typing tools. It actually reached stable performance **(Fig. 3)** when more genomes were graphed.  For both EVG and other graph-based tools, we used the same simulated and real variant dataset  and short read dataset. We detected variants by comparing multiple genomes within the previ ously published population to the reference genome [13-16], and then inserted these real vari ants into the reference genome to generate simulated genome. Therefore, even in the simulated  genome, our set of variants remains comprehensive and representative. The same simulation  process has been reported in previous studies as well [17]. The real and simulated variant data  sets are derived from the long-read mapping and the high-quality genome assembly compari sons. And similar ways of constructing the benchmark variant sets as we do have been also  used in human genetic variation studies [8, 9]. We believe that our variant dataset can be rep resentative and accurate enough for our benchmark study.

Therefore, this only indicated that the current graph-based genotyping methods did not  perform well in plants as in human genomes. And for explaining this performance difference  between human and plants, we have conducted comparisons of repetitiveness analyses with  single reference genomes and genome graphs between human and plants. These comparison  results are described in **lines 422-442** and **Figure S25**. The details of this analysis were also  described in **Response 4** at below. These analyses suggested that plant genome graph have  much more repetitive k-mers at nodes, which resulted in more alignment ambiguity, and there

fore may lead to genotyping failure.

**Response Table 1. F-scores under different number of graphed *A. thaliana* genomes based  on simulated data.**

**Response Table 2. F-scores under different number of graphed human genomes based on  real data.**

4. While I understand the complexity of the human genome is different from that of plant ge nomes, I am very surprised to see that the performances of the genotypers in all four plant  species evaluated here (Fig. 6) are substantially worse than those reported in human (Fig. 3 of  the PanGenie paper; https://www.nature.com/articles/s41588-022-01043-w). Actually, Ara bidopsis genome is much simpler than the human genome, therefore a better or comparable  performance should be expected. Furthermore, the performance of VG_Giraffe evaluated here  is also much worse than that reported in tomato, another plant species (Fig. 1c,d of  https://www.nature.com/articles/s41586-022-04808-9). This could further indicate that the var iant dataset used in the evaluation might not be representative and accurate.

Response 4: Regarding the genome size, we agree that the Arabidopsis genome is much simpler  than the human genome. However, the challenges of genotyping variant are also determined by  the content and characteristic of repetitive sequences. For both the k-mer alignment based and  read alignment-based graph genotyping tools, the increased level of repetitiveness will result  in more errors when mapping the short reads into the genome graphs, as multiple candidate  positions may have similar or even the same mapping score or priority.

To compare the repetitiveness of human and plant genomes, we first calculated the ratio  of non-unique k-mers for each of the genomes. We note that the higher ratio of unique k-mers  for a genome means the more difficult mapping the short reads into the correct genomic posi tions. All plant genomes tested in this study except the Arabidopsis, have higher ratio of non unique k-mers than the human genome even though they have much smaller size **(Fig. S25a)**. In particular, non-unique k-mers in the maize genome account for up to 68%, while the larger  human genome only contains 18% non-unique k-mer.

Apart from the intrinsic complexity of genomes, the repetitiveness at nodes (refers to se quences) of genome graph can also lead to read mapping ambiguity because more graphed  genomes may increase such repetitiveness. To check this, we further calculated the frequency of k-mers in all nodes of the genome graph for Arabidopsis, rice, maize, and human genomes.  As shown in **Figure S25b**, we found that the number of node-specific (frequency equals one)  k-mers in Arabidopsis is significantly lower (24%) than that in the human genome (62%). This  suggested that short reads from Arabidopsis may have more potential mapping positions in the genome graph, compared to the human genome. Taken together, we think mapping short reads  into the graph of multiple Arabidopsis genomes can be more challenging compared to doing  that for human genomes.

**Figure S25. The k-mer based comparisons of genome repetitiveness between plants and hu man based on single reference genomes (a) and (b) the node frequency of k-mers in the genome  graph.** Repetitiveness was assessed by calculating the ratio of non-specific k-mers to the total num ber of k-mers in the reference genome. The frequency of k-mers in nodes refers to the number of  occurrences of k-mers in all nodes of the genome graph. The variation data for *A. thaliana*, rice, and  maize were obtained from real population variation data used in this study. The human variation  data were obtained from the variants identified in 11 diploid populations in the PanGenie paper [2].  The number in parentheses represents the number of genomes used.

In the PanGenie article, it can be observed that they classified variations into two types:  simple and complex, based on the number of branches at the variant nodes. Variants with two  branches were categorized as simple, while variants with more than two branches were classi fied as complex **(Response Figure 4)**. This implies that variant nodes with a simple pattern are  easier to genotype, while those with a complex pattern, due to multiple branches, pose greater  complexity in genotyping. The genotyping F-score for complex variations is 0.4 lower com pared to simple regions **(Response Figure 5)**. This effect is particularly pronounced in repetitive  regions, where the genotyping performance is poorer **(Response Figure 5)**. In another article,  vg giraffe **[1]**, they reported a genotyping F-score range of 0.55 to 0.86, with higher scores in  variants from simple regions **(Response Figure 6c)**. As we showed above, the plant genome  graphs tend to have more such nodes with more than two branches. This can be the cause for  the issue of worse performance for PanGenie and other tools in plant genome graphs.

**Response Figure 4. Taken from the supplementary materials of the PanGenie paper [2].**

**Response Figure 5. Taken from the Fig. 3 of the PanGenie paper [2].**

**Response Figure 6. Taken from the Fig. 4 of the vg giraffe paper [1].**

Regarding the results of the pan-genome analysis of tomato [12], we carefully examined their  Method section and their Supplementary Materials. Their tests on tomato were based on simu lated data. Only for SNPs and indels genotyping, they used vg giraffe. Actually, they used Par agraph for SV genotyping. However, the detailed codes for simulating these variations, and the  specific number of variations and the graphed genomes in their test are not clear even we have  gone through the whole manuscript, supplementary documents, and their GitHub website  (https://github.com/YaoZhou89/TGG/tree/main/4.Graph_pangenome/).

Compared to that study, the F-score of SNP and indel genotyping in simulated Arabidopsis  data sets from vg giraffe is slightly lower when seven alternative genomes are graphed, but the  genotyping performance is similar to that in tomatoes when only one alternative genome is  graphed **(Response Figure 7)**. For SV genotyping using Paragraph, our tests actually presented  higher F-scores in Arabidopsis **(Response Figure 7b)** and rice, and similar F-scores in soybean  **(Fig. 1)**. However, it should be noted that the quantity of variations or the number of graphed  genomes can greatly affect the evaluation result.

**Response Figure 7. (a) Taken from the Fig. 1 of the tomato paper [12]. (b) Data from this  study. SNPs and indels were genotyped using vg giraffe, while SVs were genotyped using  Paragraph.**

5. Line 333-335, "The EVG pipeline starts with the selection of the most suitable software  according to the reference genome size, sequencing data quality, type and number of variants,  and software preference": This is the core of the EVG pipeline, while the rules for selecting the  software was not clearly described. The description in the method is also vague (Line 609-615).  A table listing which genotypers will be selected for run in EVG under different reference ge

nome sizes, sequencing data qualities/depths, type and number of variants... BTW, what is the  exact meaning of "software preference" here?

Response 5: Thank you very much for your comments. In the revised manuscript, we have  provided a detailed description of how EVG software selection was performed **(lines 657-666)**.  Software preference refers to the requirements of the software for sequencing data and variants. We have reworded this phrase to make it clearer.

The fast mode of EVG allows us to exclusively use Paragraph for genotyping SVs. In the  revised manuscript, **Figure S19** and **Table S16** illustrate the criteria for selecting software.

**Figure S19. The process of software selection in the EVG pipeline.**

6. For maize real dataset, only chr10 was analyzed according to Table S5. However, this was  not mentioned in the manuscript, and it seems the entire genome was analyzed based on the  CPU time (Fig. S19c). Please make this clear.

Response 6: Sorry for this confusion. The previous manuscript mentioned the runtime for the  entire maize genome because we conducted the testing for the whole maize genome based on  the simulated data. In the revised manuscript, we have added a number of variants in the simu lated maize genome. To complete the genotyping in limited time, we only performed the testing  on chromosome 10 of maize. Therefore, the revised manuscript now reports the runtime spe cifically for chromosome 10 of maize. We have also added labels on the figures to indicate this **(Fig. S23 and Fig. S24)**.

7. Table 1 and Table S1 are largely identical except that Table S1 contains information for four  additional genotypers. Table 1 can be removed.

Response 7: Thanks. We have now removed **Table 1** and kept the **Table S1**.

8. It would be more readable if some figures are converted to tables. Alternatively, a supple mentary table lists the precision, recall and F-measure values for all the tests would be very  helpful.

Response 8: Thank you very much for your suggestion. We have now listed all these values in  **Tables S7-S15**.

9. Line 385-386: This statement is wrong.

Response 9: Thanks. In the revised manuscript, we have corrected it.

10. It is surprising that only 5.8 million SNPs were included in the maize genome graphs based  on the simulated data (Table S4) as genomes of different maize cultivars are quite divergent.  From the real data of 7 maize lines, 1.94 million SNPs were identified on the smallest chromo some (chr10, ~7% of the maize genome; Table S5), equivalent to ~28 million in the entire  maize genome. These numbers are highly inconsistent. In addition, the numbers of SVs in all  real datasets (Table S5) are unexpectedly low.

Response 10: We agree that the number of simulated variants was smaller than the real data  sets. In the revised manuscript, we simulated much more variants for maize, where their variant  number are now close to the real data set **(Response Table 3)**. However, to finish these geno typing tests for simulated variants in limited time, we only conducted these tests on chromo some 10. Additionally, we compared the genotyping results based on the old smaller dataset  and the new dataset. Actually, as shown in **Response Table 4**, there are no big difference on  the performance of each graph-based genotyping tool, where only three cases show F-score  differences larger than 0.2.

Regarding the number of SVs in the real dataset, it is indeed lower than the true value. This  is because we want to reduce the impact of those false negative SV callings. To obtain high quality real SV datasets, we have integrated the long-read mapping-based method and the ge nome assembly alignment-based method **(lines 587-613)**. This integrating method should sig nificantly reduce the number of false positive SVs, which is also employed in many SV bench mark studies for such as human, tomato and soybean [8, 9, 12, 15], although this integrating  method can miss some real SVs only identified by single method.

**Response Table 3. The number of simulated genetic variants for maize genome.**

**Response Table 4. The genotyping result comparisons between the genotyping based on  the old smaller dataset from whole genome and the genotyping on chromosome 10 based  on the new dataset.**

**References**

1. Siren J, Monlong J, Chang X, Novak AM, Eizenga JM, Markello C, Sibbesen JA, Hickey  G, Chang PC, Carroll A, et al: **Pangenomics enables genotyping of known structural  variants in 5202 diverse genomes.** *Science* 2021, **374:**abg8871.

2. Ebler J, Ebert P, Clarke WE, Rausch T, Audano PA, Houwaart T, Mao Y, Korbel JO, Eichler  EE, Zody MC, et al: **Pangenome-based genome inference allows efficient and accurate  genotyping across a wide spectrum of variant classes.** *Nat Genet* 2022, **54:**518-525.

3. McKenna A, Hanna M, Banks E, Sivachenko A, Cibulskis K, Kernytsky A, Garimella K,  Altshuler D, Gabriel S, Daly M, DePristo MA: **The Genome Analysis Toolkit: a  MapReduce framework for analyzing next-generation DNA sequencing data.** *Genome  Res* 2010, **20:**1297-1303.

4. Poplin R, Chang P-C, Alexander D, Schwartz S, Colthurst T, Ku A, Newburger D, Dijamco  J, Nguyen N, Afshar PT, et al: **A universal SNP and small-indel variant caller using deep  neural networks.** *Nat Biotechnol* 2018, **36:**983-987.

5. Rausch T, Zichner T, Schlattl A, Stutz AM, Benes V, Korbel JO: **DELLY: structural  variant discovery by integrated paired-end and split-read analysis.** *Bioinformatics* 2012,  **28:**i333-i339.

6. Gao Y, Yang X, Chen H, Tan X, Yang Z, Deng L, Wang B, Kong S, Li S, Cui Y, et al: **A  pangenome reference of 36 Chinese populations.** *Nature* 2023, **619:**112-121. 7. Liao WW, Asri M, Ebler J, Doerr D, Haukness M, Hickey G, Lu S, Lucas JK, Monlong J,  Abel HJ, et al: **A draft human pangenome reference.** *Nature* 2023, **617:**312-324.

8. Zook JM, McDaniel J, Olson ND, Wagner J, Parikh H, Heaton H, Irvine SA, Trigg L, Truty  R, McLean CY, et al: **An open resource for accurately benchmarking small variant and  reference calls.** *Nat Biotechnol* 2019, **37:**561-566.

9. Zook JM, Hansen NF, Olson ND, Chapman L, Mullikin JC, Xiao C, Sherry S, Koren S,  Phillippy AM, Boutros PC, et al: **A robust benchmark for detection of germline large  deletions and insertions.** *Nat Biotechnol* 2020, **38:**1347-1355.

10.Shang L, Li X, He H, Yuan Q, Song Y, Wei Z, Lin H, Hu M, Zhao F, Zhang C, et al: **A  super pan-genomic landscape of rice.** *Cell Res* 2022, **32:**878-896.

11.Zhang F, Xue H, Dong X, Li M, Zheng X, Li Z, Xu J, Wang W, Wei C: **Long-read  sequencing of 111 rice genomes reveals significantly larger pan-genomes.** *Genome Res* 2022, **32:**853-863.

12.Zhou Y, Zhang Z, Bao Z, Li H, Lyu Y, Zan Y, Wu Y, Cheng L, Fang Y, Wu K, et al: **Graph  pangenome captures missing heritability and empowers tomato breeding.** *Nature* 2022,  **606:**527-534.

13.Jiao WB, Schneeberger K: **Chromosome-level assemblies of multiple Arabidopsis  genomes reveal hotspots of rearrangements with altered evolutionary dynamics.** *Nat  Commun* 2020, **11:**989.

14.Hufford MB, Seetharam AS, Woodhouse MR, Chougule KM, Ou SJ, Liu JN, Ricci WA,  Guo TT, Olson A, Qiu YJ, et al: **De novo assembly, annotation, and comparative analysis  of 26 diverse maize genomes.** *Science* 2021, **373:**655-662.

15.Liu Y, Du H, Li P, Shen Y, Peng H, Liu S, Zhou GA, Zhang H, Liu Z, Shi M, et al: **Pan Genome of Wild and Cultivated Soybeans.** *Cell* 2020, **182:**162-176 e113. 16.Song JM, Guan Z, Hu J, Guo C, Yang Z, Wang S, Liu D, Wang B, Lu S, Zhou R, et al:  **Eight high-quality genomes reveal pan-genome architecture and ecotype  differentiation of Brassica napus.** *Nat Plants* 2020, **6:**34-45.

17.Kosugi S, Momozawa Y, Liu X, Terao C, Kubo M, Kamatani Y: **Comprehensive  evaluation of structural variation detection algorithms for whole genome sequencing.** *Genome Biol* 2019, **20:**117.

**2^nd^ round**

**Reviewer 2**

Thank you for your responses to my comments, and for the changes to the paper. I consider that my comments have been properly addressed. The other reviewers also raised many good points: if the other reviewers consider that their comments were also properly addressed, then I am fine to accept the paper.

**Reviewer 4**

While the revised manuscript shows some improvements, there are still outstanding concerns that need to be addressed.

1. The authors added a figure and a table (Fig. S19 and Table S16) and a description (Line 657-671) to explain how the EVG pipeline selects different programs for genotyping. Based on this information, under the ‘precise’ mode, EVG will select programs Paragraph, BayesTyper, and vg map for species with genomes <1Gb (such as Arabidopsis and rice). However, the selection for PanGenie and GraphTyper2 based on read length and depth is confusion and inconsistent. Based on Fig. S19, PanGenie will be selected when read length is < 130 bp or read depth is < 5x, while GraphTyper will be selected when read length > 130 bp and read depth > 5x. However, based on Table S16, GraphTyper2 will be selected when read depth is < 5x. In addition, I could not find the information to support why 130 bp and 5x are used here as the cutoff values since in this study 1) only read lengths of 100 bp, 150 bp and 250 bp were evaluated, and 2) sequencing data with depth < 5x were not evaluated at all.
Line 494-495: The authors mentioned “heterozygous rate” was used in selecting the programs, but this was not reflected in the EVG pipeline.

2. Regardless, at least Paragraph, BayesTyper, and vg map were all selected for genotyping Arabidopsis and rice datasets. Then how the CPU time for EVG-precise was much less than that for one of the used programs (vg map; Fig. S23b,e,f,g) or the combined time of the two selected programs (Paragraph and vg map; Fig. S23a,d)?

3. Based on the information provided by the authors, it is still not clear to me which programs are selected under the ‘fast’ mode of EVG. Based on the text (“EVG provides a ‘fast’ model where only variants larger than 50 bp will be genotyped by Paragraph”) and Fig. S19, it seems that only Paragraph is used for large SV genotyping. Then
a) what programs are used in EVG-fast for genotyping SNPs and indels (the authors reported SNP and indel genotyping results pretty much for all their analyses while I could not find this important information)?
b) Since only Paragraph was used, then why the CPU time for EVG-fast was much less than that for Paragraph in all test cases (Fig. S23)?
c) According to Table S7-15, EVG-fast and EVG-precise had almost the identical precision, recall and F values in almost all analyses, which were higher than those of Paragraph. If only Paragraph was used in EVG-fast for genotyping large SVs, then how can EVG-fast achieve such high accuracy? Since EVG-fast and EVG-precise achieve similar accuracy, why EVG-precise is needed?

4. It is still surprising to me that including more genomes in the graph decreases (sometimes substantially) the performance of variant genotyping for most of the tools, especially in genotyping large SVs, for both plants and human (Response Tables 1 and 2). This seems very misleading. The authors’ explanation to this is that “this only indicated that the current graph-based genotyping methods did not perform well in plants as in human genomes”. However, other factors could also complicate the analysis results. These may include the quality of variants included in graph genome construction and the way to deal with multi-allelic variants. This type of information was not clearly provided in the manuscript. It seems that the authors used Assemblytics to call SVs between the alternative genomes and the reference genome (Line 553, 594, 610), which could include many false positives. In addition, the authors might cluster multi-allelic SVs (from multiple alternative genomes) into bi-allelic SVs, as they did in constructing the benchmarking SV dataset (Line 586-598) and in the EVG pipeline (Line 673-675), using positional difference of 200 bp and SV length difference ratio of 0.25 as the cutoff. This could cause problems in both read mapping and SV genotyping as the 200 bp is even longer than the Illumina reads used for genotyping. In addition, this clustering could compromise the efficiency of graph pan-genomes as quite a lot of genetic diversity in the population is lost/discarded. However, at this stage it is very hard for me to evaluate this due to the lack of the related information and the datasets. In addition, it would be great if the author can provide some summary statistics of SVs (e.g., length distribution, total size in each length range etc.) that were used in constructing graph pan-genomes, especially for Arabidopsis with different number of genomes. Nonetheless, the authors need to be very careful here.

5. The performances of all programs in genotyping variants using simulated data are much better than using real data (Table S7 vs. Fig. 6). This could indicate possible oversimplification of the simulated data that failed to capture/represent the complex nature of real plant data.

6. Table S7-S15 should be converted to a format that is easier to read. I am attaching Table S7 I prepared as one example.

7. Line 77, “the mapping accuracy of reads decreases as the number of nodes in the graph increases”: The authors cited Grytten et al. for this statement. Does the study of Grytten et al. provide any data or analysis results to support this statement?

**Authors’ response**

We greatly appreciate the valuable comments from all reviewers. To fully address their comments, we have carefully performed some additional analyses and made some revisions, with the modified and newly added content highlighted in yellow in the revised manuscript. The main changes include:

1. The Tables S7-S16 in previous response are now cited accordingly in the main text. And their ID were changed accordingly. Tables S4, Table S6 (now Table S14) and Table S16 (now Table S18) were corrected.
2. As one reviewer suggested, two supplementary tables (Tables S16 and S17) are now added to give summary statistics of the count and accumulative length of SVs across different size ranges in simulated and real data sets.
3. Some details of EVG-fast and EVG-precise modes are added in the Methods section.

Below, we provide a point-by-point response to the reviewers' comments, with responses indicated in blue.

**Reviewer #2**: Thank you for your responses to my comments, and for the changes to the paper. I consider that my comments have been properly addressed. The other reviewers also raised many good points: if the other reviewers consider that their comments were also properly addressed, then I am fine to accept the paper.

**Response:** Many thanks for your support.

**Reviewer #4**

While the revised manuscript shows some improvements, there are still outstanding concerns that need to be addressed.

**Response:** Thank you very much for your reviewing. We try to address your concerns on-by-one as shown below.

1. The authors added a figure and a table (Fig. S19 and Table S16) and a description (Line 657-671) to explain how the EVG pipeline selects different programs for genotyping. Based on this information, under the ‘precise’ mode, EVG will select programs Paragraph, BayesTyper, and vg map for species with genomes <1Gb (such as Arabidopsis and rice). However, the selection for PanGenie and GraphTyper2 based on read length and depth is confusion and inconsistent. Based on Fig. S19, PanGenie will be selected when read length is < 130 bp or read depth is < 5x, while GraphTyper will be selected when read length > 130 bp and read depth > 5x. However, based on Table S16, GraphTyper2 will be selected when read depth is < 5x. In addition, I could not find the information to support why 130 bp and 5x are used here as the cutoff values since in this study 1) only read lengths of 100 bp, 150 bp and 250 bp were evaluated, and 2) sequencing data with depth < 5x were not evaluated at all.

**Response 1a:** We apologize for the error in **Table S16**. The correct information can be found in **Figure S19**, where it shows that GraphTyper2 is selected when sequencing depth exceeds 5×. When sequencing depth is lower than 5×, PanGenie is selected. We have corrected the **Table S16 (now Table S18)** in the revision.

Currently, nearly all short-read sequencing platforms run the sequencing with a read length of 100 bp, 150 bp or 250 bp. Based on our evaluation **(Fig. S8-S10)**, GraphTyper2 shows poor performance across all types of variants at 100 bp read length, especially for insertions, but much better when the short reads 150 bp. PanGenie presented relatively stable performance when reads are only 100 bp. Consequently, we consider the read length when selecting the programs for running EVG genotyping. Here, a number between 100 and 150 was used based on our evaluation. For example, 130 was used here, but 125 or 140 are also OK since the most frequently used are 100, 150 or 250.

Furthermore, our examination **(Fig. S12-S14)** revealed that GraphTyper2 also exhibited decreased performance with 5× sequencing reads, especially compared to PanGenie. So, the sequencing depth was also considered when selecting the programs for running EVG genotyping. Additionally, we developed our EVG pipeline to do genotyping using as fewer short reads as possible. Although we have not evaluated the performance of graph-based genotypers in all ranges of sequencing depth here, we provide a choice for users when their samples are sequenced with much lower depth. Because for those large genomes such as bread wheat, sequencing depths of population-level data are often below than 5×.

Line 494-495: The authors mentioned “heterozygous rate” was used in selecting the programs, but this was not reflected in the EVG pipeline.

**Response 1b:** Thank you for pointing this out. The heterozygous rate was not considered in the current EVG pipeline. We have corrected this sentence accordingly.

2. Regardless, at least Paragraph, BayesTyper, and vg map were all selected for genotyping Arabidopsis and rice datasets. Then how the CPU time for EVG-precise was much less than that for one of the used programs (vg map; Fig. S23b,e,f,g) or the combined time of the two selected programs (Paragraph and vg map; Fig. S23a,d)?

**Response 2:** **Figure S19** illustrates the workflow of the EVG automated selection software. However, EVG also allows users to select genotyping software for initial running. In our testing, we found that there was not a significant difference in performance between the vg map and the vg giraffe, but the vg giraffe is much faster than the vg map. Therefore, for testing EVG, we actually used the vg giraffe instead of the vg map. As a result, the runtime of EVG-precise was significantly shorter compared to the vg map. In Figure S23 and S24, we have also included the resource consumption of EVG when vg map is used for genotyping.

3. Based on the information provided by the authors, it is still not clear to me which programs are selected under the ‘fast’ mode of EVG. Based on the text (“EVG provides a ‘fast’ model where only variants larger than 50 bp will be genotyped by Paragraph”) and Fig. S19, it seems that only Paragraph is used for large SV genotyping. Then

a) what programs are used in EVG-fast for genotyping SNPs and indels (the authors reported SNP and indel genotyping results pretty much for all their analyses while I could not find this important information)?

**Response 3a:** Sorry for this confusion. We have revised the Method section (in yellow highlight) to make it more clear. Apart from Paragraph, all other programs, selected based on the genome size, read length, and sequencing depth, were used for SNPs and indels genotyping in EVG-fast. Because Paragraph generates three files per variant site, it will require substantial disk I/O and prolonged runtime when genotyping a large number of SNPs and indels. Therefore, Paragraph is not used for SNP and indel genotyping in EVG-fast.

b) Since only Paragraph was used, then why the CPU time for EVG-fast was much less than that for Paragraph in all test cases (Fig. S23)?

**Response 3b:** In EVG-fast mode, Paragraph is only used for genotyping structural variants. And because Paragraph takes much more time for genotyping SNPs and indels compared to other genotyping programs, EVG-fast can be much faster than Paragraph (for all types of variants).

c) According to Table S7-15, EVG-fast and EVG-precise had almost the identical precision, recall and F values in almost all analyses, which were higher than those of Paragraph. If only Paragraph was used in EVG-fast for genotyping large SVs, then how can EVG-fast achieve such high accuracy? Since EVG-fast and EVG-precise achieve similar accuracy, why EVG-precise is needed?

**Response 3c:** In the EVG-fast mode, Paragraph is exclusively used for genotyping structural variants, while the remaining selected tools are still utilized for genotyping all types of variants. Therefore, multiple programs are actually used for genotyping structural variants in EVG-fast, which can improve the genotyping performance compared to only running Paragraph.

As the tool Paragraph was included for genotyping SNPs and indels in EVG-precise, EVG-precise presented relatively higher performance than EVG-fast in some analyses. For example, in real maize data, the F-score of EVG-precise for SNPs and Indels are 0.67 and 0.72, respectively, whereas EVG-fast achieves only 0.48 and 0.54 for these variants. So, we included the Paragraph for genotyping SNPs and indels in EVG-precise.

4. It is still surprising to me that including more genomes in the graph decreases (sometimes substantially) the performance of variant genotyping for most of the tools, especially in genotyping large SVs, for both plants and human (Response Tables 1 and 2). This seems very misleading. The authors’ explanation to this is that “this only indicated that the current graph-based genotyping methods did not perform well in plants as in human genomes”. However, other factors could also complicate the analysis results. These may include the quality of variants included in graph genome construction and the way to deal with multi-allelic variants. This type of information was not clearly provided in the manuscript. It seems that the authors used Assemblytics to call SVs between the alternative genomes and the reference genome (Line 553, 594, 610), which could include many false positives. In addition, the authors might cluster multi-allelic SVs (from multiple alternative genomes) into bi-allelic SVs, as they did in constructing the benchmarking SV dataset (Line 586-598) and in the EVG pipeline (Line 673-675), using positional difference of 200 bp and SV length difference ratio of 0.25 as the cutoff. This could cause problems in both read mapping and SV genotyping as the 200 bp is even longer than the Illumina reads used for genotyping. In addition, this clustering could compromise the efficiency of graph pan-genomes as quite a lot of genetic diversity in the population is lost/discarded. However, at this stage it is very hard for me to evaluate this due to the lack of the related information and the datasets. In addition, it would be great if the author can provide some summary statistics of SVs (e.g., length distribution, total size in each length range etc.) that were used in constructing graph pan-genomes, especially for Arabidopsis with different number of genomes. Nonetheless, the authors need to be very careful here.

**Response 4:** We fully agree that the quality of variants and improbable clustering of multi-allelic SVs may also affect the genotyping performance. However, we would like to point out that the genetic variants for the **Response Table 2** were actually obtained from the PanGenie paper [1]. Therefore, the same phenomenal that including more genomes in the graph decreases the performance of variant genotyping for most of the tools was also observed, even though a different way of constructing the variant data set was used.

But for the SV calling, we actually integrated both the assembly comparison-based method (using Assemblytics) and the long read mapping-based method (Sniffles) **(Lines 592-600)**. The resulting SVs shared by Sniffles and Assemblytics were used for genotyping. So, we believe that the false positive rate of our SV data set should be much lower than that resulting from Assemblytics only. This integrating strategy for SV calling has also been employed in some benchmarking studies or tool performance evaluation [2, 3].

Furthermore, for the SV clustering, the processes described in **lines 586-598** and **lines 673-675 (now lines 686-690)** do not involve merging alleles. Regarding **lines 586-598**, we only identified those SVs both called by the tool Assemblytics and Sniffles. This processing allows us to identify high-confidence variants. We repeated this process for each sample and used VCFtools to merge the variants across the population variants. Notably, this VCFtools merging process did not remove multi-allelic SV. Only for those identical alleles from different samples, one was kept in the resulting VCF file. But for those different alleles, for example, two or more insertions at the same reference genomic positions from different samples will still be kept. Therefore, no genetic diversity in the population is lost or discarded here.

Regarding **lines 673-675**, here we only did SV clustering for genotyping resulting from different programs for each sample. We have not done clustering SV from different samples. For each sample, the input SVs to be genotyped are the same. But some graph-based programs may output different coordinates for some SVs. For example, variant coordinates resulted from vg genotyping may have some changes. Here, our clustering process aims to determine the true coordinates of the output SVs which they should be correspond to in the input SVs. Therefore, this process also does not involve multi-allelic merging.

As you suggested, we tabulated the counts and lengths of different types of variants in **Tables S16 and S17**. Additionally, **Response Figs. 1-3** displays the number of variants in the *Arabidopsis* genome graph. Both the size distribution of different types of SV and the count of different size range in *A. thaliana* are similar with what we previously found in real *A. thaliana* genomes [4].Notably, for SVs in the same genomic position and found in multiple genomes, only one was counted.

**Response Figure 1. Distribution of variants (deletions and insertions) across different size ranges in genome graphs containing different numbers of genomes.**

**Response Figure 2. Distribution of variants (inversions) across different size ranges in genome graphs containing different numbers of genomes.**

**Response Figure 3. Distribution of variants (duplications) across different size ranges in genome graphs containing different numbers of genomes.**

5. The performances of all programs in genotyping variants using simulated data are much better than using real data (Table S7 vs. Fig. 6). This could indicate possible oversimplification of the simulated data that failed to capture/represent the complex nature of real plant data.

**Response 5**: The performance of genotyping programs can be benchmarked using simulated and/or real data. The simulated data can provide the desired number of variants, sequencing depth and length, deviation of SV breakpoints, etc. We used simulated data to figure out how well these genotyping programs perform in different scenarios in terms of variant type and size, sequencing parameters, genomic context, and complexity, as well as graph size.

Here, we used the same methods to generate the simulated data as other benchmarking studies often do. And the simulated data are mostly derived from previously published variant data sets. According to our evaluation, the general differences among different genotyping tools in simulated data are as similar as those in real data. By the way, we would like to point out that the performances using simulated data are frequently better than the real data in many benchmarking studies.

Besides, the breakpoints of the simulated variants are accurate **(Table S5)**. However, the real variant data sets **(Fig. 6)** were obtained based on long-read mappings and assembly comparisons. Although we have used hybrid approaches to construct the variant data set, some residual imprecise breakpoints may affect the genotyping performance of some graph-based genotypers, especially those less tolerant of inaccurate SV breakpoints. We actually evaluated the impact of imprecise breakpoints on SV genotyping **(Fig. 4a and Fig. S16)** and indeed found that some graph-based tools were very sensitive to the accuracy of SV breakpoints. However, our EVG pipeline can alleviate this problem and showed much better performance compared to a single genotyping tool. Therefore, for the individual genotyping tool, it showed better performance in simulated data.

6. Table S7-S15 should be converted to a format that is easier to read. I am attaching Table S7 I prepared as one example.

**Response 6:** Thank you very much for your good suggestion. We have reformatted **Tables S7-S15** **(now Tables S5-S13)** based on your attached file.

7. Line 77, “the mapping accuracy of reads decreases as the number of nodes in the graph increases”: The authors cited Grytten et al. for this statement. Does the study of Grytten et al. provide any data or analysis results to support this statement?

**Response 7:** Grytten et al., cited the reference “Garrison et al. Variation graph toolkit improves read mapping by representing genetic variation in the reference. Nat. Biotechnol. 36, 875–879 (2018).” to support a statement “As complex graphs containing many genetic variants can result in long indexing time as well as poor mapping accuracy” in the Background of their study. Here at **Line 78**, we actually would like to express a similar opinion. We have revised this statement and cited the original study of Garrison et al. in our revised manuscript.

**References**

1.	Ebler J, Ebert P, Clarke WE, Rausch T, Audano PA, Houwaart T, Mao Y, Korbel JO, Eichler EE, Zody MC, et al: **Pangenome-based genome inference allows efficient and accurate genotyping across a wide spectrum of variant classes.** *Nat Genet* 2022, **54:**518-525.

2.	Kosugi S, Momozawa Y, Liu X, Terao C, Kubo M, Kamatani Y: **Comprehensive evaluation of structural variation detection algorithms for whole genome sequencing.** *Genome Biol* 2019, **20:**117.

3.	Zook JM, Hansen NF, Olson ND, Chapman L, Mullikin JC, Xiao C, Sherry S, Koren S, Phillippy AM, Boutros PC, et al: **A robust benchmark for detection of germline large deletions and insertions.** *Nat Biotechnol* 2020, **38:**1347-1355.

4.	Jiao WB, Schneeberger K: **Chromosome-level assemblies of multiple Arabidopsis genomes reveal hotspots of rearrangements with altered evolutionary dynamics**. *Nat Commun* 2020, **11**:989.

**3^rd^ round**

**Reviewer 4**

Authors’ response, “In our testing, we found that there was not a significant difference in performance between the vg map and the vg giraffe, but the vg giraffe is much faster than the vg map”: then why is vg map used in EVG for genomes sizes < 1GB instead of vg giraffe (Line 668-669, Table S18, Fig. S19)? The authors’ results (Table S5 and Fig. 6) also do not seem to support this selection.

Based on the authors’ description, the only difference between EVG-fast and EVG-precise is that in EVG-fast, Paragraph is only used for genotyping SVs, while in EVG-precise, Paragraph is used for genotyping all variants (SVs, SNPs, indels). The same other programs are used in these two modes, e.g., BayesTyper, vg map or vg giraffe, and PanGenie and/or GraphTyper2. Therefore, for SV genotyping, EVG-fast and EVG-precise employ the exact same programs. Then why are there differences between the results of EVG-fast and EVG-precise for genotyping deletions and insertions (e.g., rows 16-21 of Table S6), although the differences are small?

Line 77, “the mapping accuracy of reads decreases as the number of nodes (variants) in the graph increases”: In the authors’ response, they indicated that they would like to express a similar opinion to the statement “As complex graphs containing many genetic variants can result in long indexing time as well as poor mapping accuracy” described in Grytten et al. However, these two statements convey entirely different meanings. Grytten et al., along with Garrison et al., emphasize that “the most complex regions” (such as highly repetitive regions) in the graph could lead to poor mapping. This is not directly related to “the number of nodes”. In addition, upon reviewing Garrison et al., I couldn’t find any data or analysis results supporting the claim that “the mapping accuracy of reads decreases as the number of nodes (variants) in the graph increases”.

Line 349: Is the “number of variants” used as a selection criterion for EVG? Couldn’t find this in Table S18 and Fig. S19.

**Authors’ response**

1. Authors’ response, “In our testing, we found that there was not a significant

difference in performance between the vg map and the vg giraffe, but the vg giraffe is

much faster than the vg map”: then why is vg map used in EVG for genomes sizes <

1GB instead of vg giraffe (Line 668-669, Table S18, Fig. S19)? The authors’ results

(Table S5 and Fig. 6) also do not seem to support this selection.

Response 1: In our testing, vg map and vg giraffe have no significant differences in

terms of genotyping precision and recall. However, vg map is theoretically better than

vg giraffe ac-cording to the previous findings of their developers. Because vg giraffe is

much faster than vg map, it should be more suitable for larger genomes (more variants

or complexed genome graphs are expected, compared to smaller genomes).

Therefore, we choose vg map for ge-nomes smaller than 1GB and vg giraffe for larger

genomes (> 1 Gb).

2. Based on the authors’ description, the only difference between EVG-fast and EVG-

precise is that in EVG-fast, Paragraph is only used for genotyping SVs, while in EVG-

precise, Paragraph is used for genotyping all variants (SVs, SNPs, indels). The same

other programs are used in these two modes, e.g., BayesTyper, vg map or vg giraffe,

and PanGenie and/or GraphTyper2. Therefore, for SV genotyping, EVG-fast and EVG-

precise employ the exact same programs. Then why are there differences between the

results of EVG-fast and EVG-precise for geno-typing deletions and insertions (e.g.,

rows 16-21 of Table S6), although the differences are small?

Response 2: For BayesTyper2, the results of multiple runs with the same dataset can

show some differences. This might be due to the employment of Bloom Filters, a data

structure known to have a certain rate error, which can lead to slight discrepancies in

the results. Therefore, the results of BayesTyper for EVG-precise and EVG-fast modes

are not always 100% identical. Moreover, even with the same sequencing data, the

result from Paragraph with all types of variants (EVG-precise) can also have small

differences with the result from Paragraph with only structural variations (EVG-fast). It

seems that the genotyping of smaller variants may impact the genotyping of SV when

all of them are loaded for genotyping when using Paragraph.

3. Line 77, “the mapping accuracy of reads decreases as the number of nodes

(variants) in the graph increases”: In the authors’ response, they indicated that they

would like to express a similar opinion to the statement “As complex graphs containing

many genetic variants can result in long indexing time as well as poor mapping

accuracy” described in Grytten et al. However, these two statements convey entirely

different meanings. Grytten et al., along with Garrison et al., emphasize that “the most

complex regions” (such as highly repetitive regions) in the graph could lead to poor

mapping. This is not directly related to “the number of nodes”. In addition, upon

reviewing Garrison et al., I couldn’t find any data or analysis results sup-porting the

claim that “the mapping accuracy of reads decreases as the number of nodes

(variants) in the graph increases”.

Response 3: As the Supplementary Figure 1 of this vg paper (Garrison et al., 2018)

indicates, from top to bottom, the alignment performance for allele frequency

thresholds of 0, 0.001, 0.01, and 0.1, are getting better. Obviously, the lower the allele

frequency threshold, the more variants are included in genome graphs. It is evident

that as the number of variants increases, the false positive rate of alignments also

increases based on this figure. Therefore, we think this can support the claim that the

more nodes there are, the lower the accuracy of the alignment. We now tone down this

sentence accordingly, as the vg authors haven’t emphasized this claim.

4. Line 349: Is the “number of variants” used as a selection criterion for EVG? Couldn’t

find this in Table S18 and Fig. S19.

Response 4: Sorry for the confusion. The “number of variants” is not used as a

selection cri-terion for EVG. We revised accordingly.
